# AKT-induced lncRNA VAL promotes EMT-independent metastasis through diminishing Trim16-dependent Vimentin degradation

**DOI:** 10.1038/s41467-020-18929-0

**Published:** 2020-10-12

**Authors:** Han Tian, Rong Lian, Yun Li, Chenying Liu, Shujun Liang, Wei Li, Tianyu Tao, Xingui Wu, Yaokai Ye, Xia Yang, Jian Han, Xuwei Chen, Jun Li, Yukai He, Mengfeng Li, Jueheng Wu, Junchao Cai

**Affiliations:** 1 Department of Microbiology, Zhongshan School of Medicine, Sun Yat-sen University, Guangzhou, 510080 Guangdong China; 2 Institute of Tissue Transplantation and Immunology, Department of Immunobiology, Jinan University, Guangzhou, 510632 Guangdong China; 3 Department of Biochemistry, Zhongshan School of Medicine, Sun Yat-sen University, Guangzhou, 510080 Guangdong China; 4 Clinical Medicine, Zhongshan School of Medicine, Sun Yat-sen University, Guangzhou, 510080 Guangdong China; 5 Cancer Institute, Southern Medical University, Guangzhou, 510515 Guangdong China; 6 Department of Medicine and Georgia Cancer Center, Medical College of Georgia, Augusta University, Augusta, GA 30912 USA; 7 Department of Immunology, Zhongshan School of Medicine, Sun Yat-sen University, Guangzhou, 510080 Guangdong China; 8 Key Laboratory of Tropical Disease Control, Ministry of Education, Sun Yat-sen University, Guangzhou, 510080 China

**Keywords:** Non-small-cell lung cancer, Metastasis

## Abstract

Despite the importance of AKT overactivation in tumor progression, results from clinical trials of various AKT inhibitors remain suboptimal, suggesting that AKT-driven tumor metastasis needs to be further understood. Herein, based on long non-coding RNA (lncRNA) profiling induced by active AKT, we identify that VAL (Vimentin associated lncRNA, LINC01546), which is directly induced by AKT/STAT3 signaling, functions as a potent pro-metastatic molecule and is essential for active AKT-induced tumor invasion, metastasis and anoikis resistance in lung adenocarcinoma (LAD). Impressively, chemosynthetic siRNAs against VAL shows great therapeutic potential in AKT overactivation-driven metastasis. Interestingly, similar to activated AKT in LAD cells, although unable to induce epithelial-mesenchymal transition (EMT), VAL exerts potent pro-invasive and pro-metastatic effects through directly binding to Vimentin and competitively abrogating Trim16-depedent Vimentin polyubiquitination and degradation. Taken together, our study provides an interesting demonstration of a lncRNA-mediated mechanism for active AKT-driven EMT-independent LAD metastasis and indicates the great potential of targeting VAL or Vimentin stability as a therapeutic approach.

## Abstract (web summary)

The role of long non-coding RNA (lncRNA) in AKT-driven tumor development is unclear. Here, the authors identify VAL (Vimentin associated lncRNA) to be directly induced by AKT/STAT3 signaling and report a lncRNA-mediated mechanism for active AKT-driven EMT-independent lung adenocarcinoma metastasis.

## Introduction

Lung cancer is the most commonly diagnosed cancer type and the leading cause of cancer-related death worldwide^1^. Non-small-cell lung carcinoma (NSCLC) accounts for ~85% of all lung cancer cases, and lung adenocarcinoma (LAD) is a predominant histological type, which constitutes around 50% of NSCLC cases^2^. It is estimated that nearly two-thirds of NSCLC patients show evidence of local or distant metastasis at initial diagnosis, whose median survival is ~7 months, and as low as 1% of patients with metastatic NSCLC survive for 5 or more years after the diagnosis of metastases^3^. NSCLC cells in primary tumor lesion often spread to lymph nodes, contralateral lung, and distant organs such as bones, brain, and liver, which usually displays extremely low responsiveness to the initial anti-cancer treatment and fairly high frequencies of posttreatment relapse^4^. It is therefore important to better understand the mechanisms underlying NSCLC metastasis and identify effective therapeutic targets or prognostic biomarkers for metastatic NSCLC.

Tumor invasion and metastasis involve a sophisticated cascade process, including acquisition of invasive and migratory abilities, detachment from the primary tumor and breach of the basement membrane, intravasation, survival in circulation flow, extravasation, adhesion, and colonization at the secondary site^5^. It is important to note that epithelial-mesenchymal transition (EMT), wherein epithelial cells lose polarization and cell-and-cell contacts, and gain a fibroblast-like morphology, has been shown to be a critical first step for the metastatic cascade process^6^. Undergoing EMT usually empowers non- or low-metastatic tumor cells to be highly aggressive, and lost expression of epithelial markers such as E-cadherin and increased expression of mesenchymal markers such as Vimentin can be detected in, at least a subset of metastatic tumor tissues^7^. Nevertheless, metastasis from primary tumors also often occurs in the absence of EMT activation. Specifically, Fischer et al. recently showed that using an EMT lineage-tracing system in spontaneous breast-to-lung metastasis model, although a small proportion of tumor cells within primary breast tumors undergo EMT, spontaneous lung metastases mainly consist of non-EMT tumor cells with epithelial phenotype^8^. These studies have highlighted the fact that the mechanisms underlying tumor metastasis are far more complex than what has been understood.

The AKT signaling pathway plays pivotal roles in the development of almost all hallmarks of cancer cells, mechanistically through a wide variety of downstream effectors during tumor development and progression^9,10^. The close correlation of AKT overactivation with clinical staging as well as poor patient prognosis is well documented in various types of cancers, including NSCLC^11,12^. Mounting evidence have revealed various mechanisms via which AKT overactivation promotes tumor invasion and metastasis. For example, not only can active AKT proteins activate the pro-EMT transcriptional factors, directly or indirectly, to stimulate EMT program, but also induce expression of many pro-invasive or pro-metastatic molecules without conferring EMT phenotype, leading to tumor metastasis^13–16^. Notably, aberrant AKT activities are also often found in epithelial cells with no or low-metastatic ability, suggesting that AKT-driven tumor metastasis might be quite dependent on molecular scenario^17,18^. Despite the importance of activated AKT in pan-cancers and tremendous efforts to develop AKT-targeted therapies, results from clinical trials of various AKT inhibitors remain suboptimal, even in combination with inhibitors targeting its downstream protein effectors, suggesting that AKT-driven tumor metastasis needs to be further investigated and understood.

Long non-coding RNAs (lncRNAs), a class of endogenous RNAs longer than 200 nucleotides with extremely low protein-coding abilities, have been shown to be essentially involved in a variety of fundamental biological events and diseases^19,20^. At the molecular level, lncRNAs can act as decoys to bind to proteins and microRNAs, or scaffolds or guides to regulate protein–protein or protein–DNA interactions and thus can regulate gene expression at epigenetic, transcriptional, translational, posttranscriptional, and posttranslational levels^21–25^. Mounting evidence suggests that lncRNAs represent a unique class of master regulators in tumor development and progression. For example, an NF-κB Interacting LncRNA binds to the NF-κB/IκB complex and inhibits NF-κB signaling by masking the phosphorylation motifs of IκB and stabilizing the complex, leading to suppression of breast cancer metastasis^26^. However, the roles of the large majority of lncRNAs in AKT-driven tumor development and progression remain unclear.

In this current study, we profile global lncRNA expression in activated AKT-overexpressing LAD cell lines with significantly potentiated metastatic abilities irrelevant to EMT process and identify an intergenic cytoplasmic lncRNA VAL (LINC01546, ENSG00000228459), which is induced by activated AKT via STAT3 transcriptional activity and correlates with disease progression and poor outcomes of LAD, as a potent pro-metastatic molecule. We further demonstrate that this lncRNA is essential for LAD metastasis driven by AKT overactivation. Interestingly, although unable to induce EMT, VAL potentiates LAD invasion and metastasis through directly binding to Vimentin and competitively abrogating Vimentin degradation induced by its previously unestablished E3 ligase Trim16. These results should provide insights into the metastatic process of LAD and opportunities for LAD diagnosis and treatment.

## Results

### AKT/STAT3-induced VAL correlates with poor prognosis of LAD

**Fig. 1:**
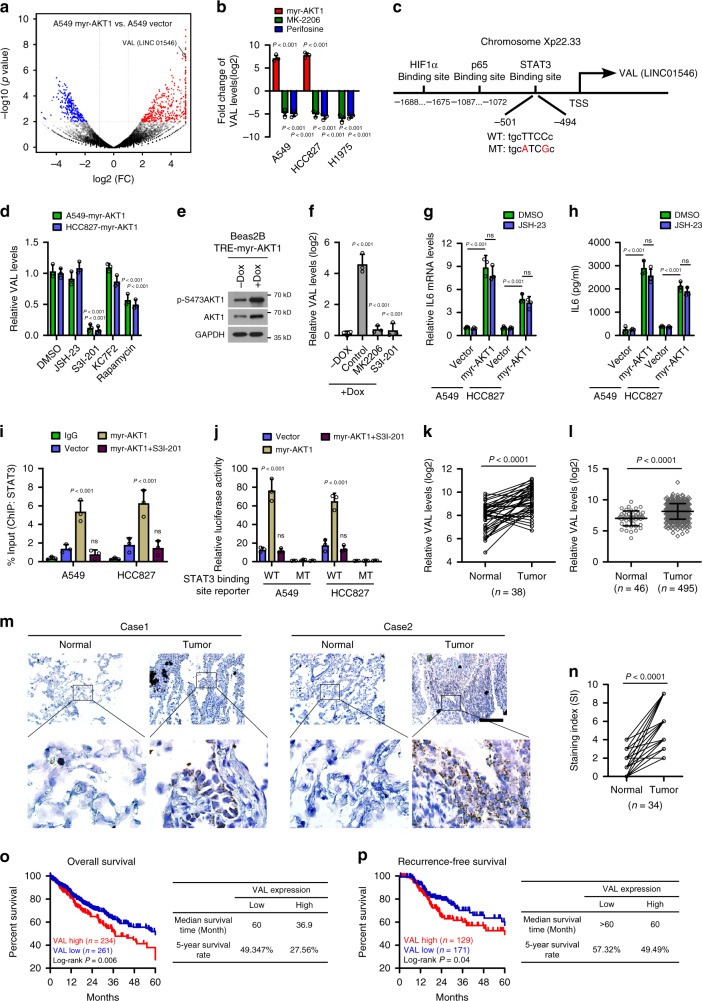
AKT/STAT3-induced VAL correlates with poor prognosis of LAD. **a** Volcano plot shows significantly upregulated (red) and down-regulated (blue) lncRNAs in A549-myr-AKT1 cells as compared to A549-vector cells based on RNA sequencing. **b** The effect of overexpressing myr-AKT1 or distinct AKT inhibitors on VAL expression. **c** Schematic diagram of predicted binding sites for STAT3, p65, and HIF1α in the promoter/enhancer region of the *VAL* (*LINC01546)* gene. The predicted binding site containing wild-type or mutated binding motif for STAT3 is shown. **d** The effect of treatment of inhibitors for p65 (JSH-23), STAT3 (S3I-201), HIF1α (KC7F2), or mTOR (Rapamycin) on VAL expression. **e**, **f** Beas2B cells were transduced with the pLVX-TRE3G-myr-AKT1 Tet-on inducible system and stimulated with 2 μg/ml doxycycline (Dox) for 48 h, followed by treatment of AKT or STAT3 inhibitor. Expression of AKT1 and VAL in the indicated cells is shown. **g**, **h** The effect of myr-AKT1 overexpression followed by p65 inhibitor treatment on IL6 expression and secretion. **i** ChIP assays show the binding of STAT3 to the predicted promoter/enhancer region of the *VAL* gene in response to the indicated treatments. **j** Relative luciferase activities of the reporter constructs spanning wild-type or mutant predicted putative binding site for STAT3. **k**, **l** Analysis of VAL expression in 38 pairs of human LAD tissue (Tumor) and adjacent non-tumor tissue (Normal), and 46 cases of lung tissue and 495 cases of LAD tissue in the TCGA LAD datasets. **m**, **n** The expression levels of VAL in 34 pairs of LAD tissue and adjacent non-tumorous tissue using RNA in situ hybridization and representative images of VAL staining are shown. Scale bar: 100 μm. **o**, **p** Kaplan–Meier analysis (Log-rank test) of the 5-year overall survival (left panel) and recurrence-free survival (right panel) of LAD patients in the TCGA LAD datasets, who were divided into low or high VAL expression subgroups. Error bars represent the means ± SD derived from three independent experiments. Statistical analyses were performed by two-way ANOVA multiple comparison analysis (**f**–**j**), two-tailed unpaired Student’s *t* test (**b**, **d**, **l**), and two-tailed paired Student’s *t* test (**k**, **n**). n.s., not significant. Source data are provided as a Source Data file.

We began this study by transducing two LAD cell lines A549 and HCC827 with a constitutively active AKT1 (myr-AKT1) construct, which failed to induce EMT phenotypic and molecular changes but greatly potentiated invasive abilities (Supplementary Fig. 1a–d). To identify lncRNA species involved in mediating active AKT-induced aggressiveness, myr-AKT1-overexpressing LAD cells and their corresponding control cells were profiled for global lncRNA expression. Among the most upregulated transcripts induced by active AKT, VAL (LINC01546, ENSG00000228459) expression was also drastically reduced by treatment with AKT inhibitors MK-2206 or Perifosine in various LAD cells (Fig. 1a, b and Supplementary Fig. 1e and Supplementary Data 1). To further reveal how active AKT induces VAL expression, genomic sequences of the promoter/enhancer regions of *VAL* locus were analyzed, and putative potential binding sites for several transcriptional factors downstream to AKT activation, including HIF1α, p65, and STAT3 were individually predicted in the promoter/enhancer regions upstream of the transcription start site of *VAL* (Fig. 1c). Moreover, treatment with inhibitors of STAT3, but not those of HIF1α or p65, significantly reversed active AKT-induced VAL expression, while mTOR inhibitor mildly decreased VAL expression in LAD cells overexpressing constitutively active AKT (Fig. 1d). Consistently, using a Tet-on inducible system in Beas2B cells, addition of doxycycline (Dox) induced myr-AKT1 expression and markedly upregulated VAL expression, and further treatment with AKT or STAT3 inhibitors significantly reversed Dox-induced VAL expression (Fig. 1e, f). Notably, transient stimulation of A549 and HCC827 cells with recombinant insulin or FGF2, which markedly activated AKT1 but slightly activated STAT3 in these LAD cells, mildly induced VAL expression whereas inhibitors of AKT, STAT3, or mTOR could completely abrogate the promoting effect of insulin or FGF2 stimulation on VAL expression (Supplementary Fig. 1f–h), indicating that activated AKT stimulates STAT3 activity by distinct pathways. Interestingly, we found that IL6 was significantly upregulated in LAD cells overexpressing constitutively active AKT, and that p65 inhibitor failed to reverse the promoting effect of constitutively active AKT on IL6 expression (Fig. 1g, h), whereas insulin or FGF2 stimulation barely affected IL6 expression in the LAD cells (Supplementary Fig. 1g). As expected, stimulation of LAD cells with recombinant IL6 protein also significantly induced VAL expression, and STAT3 inhibitor greatly reversed, while AKT inhibitor slightly compromised IL6-induced VAL expression (Supplementary Fig. 1i). In parallel, both IL6 stimulation and overexpression of constitutively active STAT3 reversely upregulated VAL expression in H1975 cells silenced for AKT1 by Dox addition using the Tet-on inducible system (Supplementary Fig. 1j, k). These data suggest that constitutively activated AKT promotes high STAT3 activity through multiple pathways, at least including NF-κB-independent IL6 transcription and mTOR pathways, to induce high levels of VAL in LAD cells. Furthermore, ChIP analysis revealed that binding of STAT3 to its predicted binding site in the upstream promoter region of *VAL* gene was much more enriched in myr-AKT1-overexpressing LAD cells, which could be completely abrogated by treatment with the STAT3 inhibitor S3I-201 (Fig. 1i). Additionally, only the luciferase activity of the reporter containing the putative STAT3 binding site with wild-type sequences was significantly increased in myr-AKT1-overexpressing LAD cells, and inhibiting STAT3 abrogated the above transcription-enhancing effect of active AKT1 (Fig. 1j), suggesting that the *VAL* gene is indeed under direct transcriptional induction by STAT3 and thus a downstream target gene of the AKT/STAT3 signaling pathway.

We then used 5′ and 3′ rapid-amplification of cDNA ends assays to analyze full-length VAL and obtained the 687 nt polynucleotide molecule, which was further found to possess the 5′-terminal cap and 3′-terminal poly (A) structures (Supplementary Fig. 2a–d). In addition, the codon substitution frequency (CSF) analysis with PhyloCSF program and WB analysis of HA expression of a construct containing full-length VAL tagged with HA in the 3′-terminal suggested extremely low protein-coding ability of the full-length VAL (Supplementary Fig. 2e, f). More importantly, we found that VAL was significantly upregulated in LAD tissues as compared with the normal tissue specimens collected in the TCGA LAD datasets, which include 38 pairs of lung cancer/normal tissues and 495 cases of lung cancer vs 46 cases of unpaired normal lung tissue, as well as in a panel of LAD cell lines as compared to an immortalized normal lung epithelial cell line Beas2B (Fig. 1k, l and Supplementary Fig. 2g). Consistently, RNA in situ hybridization confirmed overexpression of VAL in 34 cases of paraffin-embedded LAD specimens paired with adjacent non-cancerous tissues in our own collection, and also clearly revealed the cytoplasmic localization of VAL (Fig. 1m, n). In parallel, absolute quantification by real-time RT-PCR showed that the absolute VAL quantities were significantly higher in LAD tissue of advanced-stage disease (~206–975 copies per cell) than those in normal lung epithelial cells/tissue (~2–8 copies) or early-stage LAD tissue (54–188 copies) (Supplementary Table 1). Notably, the quantity of VAL in a stage III LAD tissue carrying the activating *PIK3CA* (E545K) mutation was similar to the absolute VAL level in LAD cells overexpressing myr-AKT1 (Supplementary Table 1). Moreover, using the TCGA LAD datasets, Kaplan–Meier analysis showed that LAD patients bearing high VAL expression in their lung tumors displayed a significantly shorter median survival time of 36.9 months and lower 5-year survival rate of 27.56%, compared with the 60.0-month median survival and 49.34%-rate 5-year survival for those with low VAL expression; in parallel, high VAL levels negatively correlates with short recurrence-free survival (Fig. 1o, p), suggesting that aberrant VAL expression induced by AKT/STAT3 activation might play important roles during LAD development and progression.

### VAL potently promotes invasion and metastasis in LAD

**Fig. 2:**
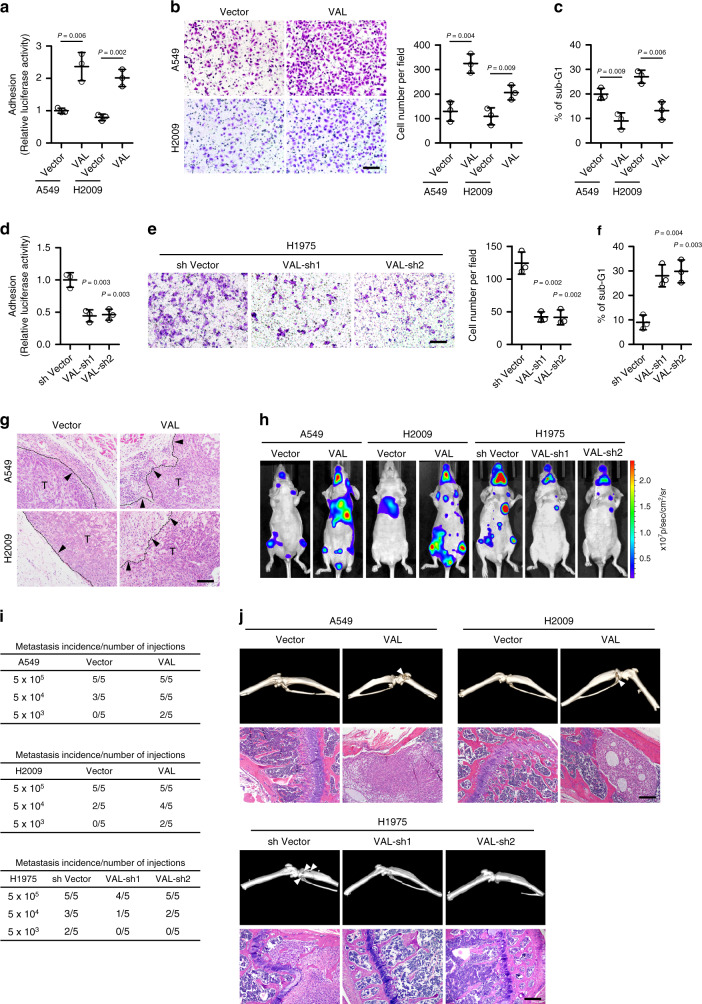
VAL potently promotes invasion and metastasis in LAD. **a** Relative luciferase activities of vector-control or VAL-overexpressing LAD cells adhering to the Matrigel. **b** Representative images and quantification of invading cells in five random fields of Matrigel-coated transwell assay. **c** The sub-G1 DNA contents of detached cells for vector-control or VAL-overexpressing A549 or H2009 cells are shown in the anoikis assay. **d**–**f** The effect of silencing VAL on cell adhesion, invasion, and suspension-induced anoikis in H1975 cells. **g** Representative images of H&E staining display a clear boundary and irregular invasive front between the dermal tissue and tumor tissue in the subcutaneous tumors (*n* = 5 per group), respectively, xenografted with vector-control and VAL-overexpressing LAD cells. Black arrows mark the direction of tumor invasion with invasive front marked by dashed line. **h** LAD cells overexpressing VAL or silenced with VAL or vector-control cells labeled with luciferase expression were injected via cardiac ventricle into nude mice (*n* = 5 per group). Representative bioluminescent images of systemic metastasis are shown. **i** Frequencies of formation of metastatic lesions in nude mice (*n* = 5 per group) intracardially injected with different cell numbers of vector-controls, VAL-overexpressing, or VAL-silenced LAD cells. **j** Representative images of metastatic bone lesions in mice (*n* = 5 per group) intracardially injected with the indicated cells were confirmed by micro-CT imaging and H&E staining. Scale bar: 100 μm (**b**, **e**, **g**, **j**). Scatter dot plots represent the means ± SD derived from three independent experiments. Statistical analyses were performed by two-tailed unpaired Student’s *t* test (**a**–**f**). Source data are provided as a Source Data file.

Next, we overexpressed VAL in A549 and H2009 cells, which express moderate levels of VAL, and silenced VAL in a LAD cell line H1975 carrying the activating *PIK3CA* (G118D) mutation and expressing high levels of VAL in order to evaluate the biological function of VAL. Of note, the absolute VAL levels in VAL-overexpressing LAD cells were within the range of those in human LAD tissue samples (Supplementary Table 1). Although VAL overexpression or knockdown failed to induce phenotypic and molecular changes of EMT (Supplementary Fig. 3a–f), LAD cells overexpressing VAL exhibited much stronger abilities to bind to and invade through the extracellular matrix, as well as to resist suspension-induced anoikis, which has been implicated to importantly contribute to tumor cell survival in the cascaded metastasis process^27,28^, as compared to vector-control cells (Fig. 2a–c). By contrast, silencing VAL in H1975 cells markedly impaired cell adhesion, invasion, and anoikis resistance (Fig. 2d–f). In consistence with these pro-metastatic effects of VAL in vitro, overexpression of VAL potently conferred low-metastatic LAD cells the invasive ability to penetrate into the neighboring subcutaneous tissue in vivo, which was barely observed in the similarly sized tumor xenografts formed by vector-control cells (Fig. 2g and Supplementary Fig. 3g, h). Moreover, when injected intracardially, as few as 5 × 10^3^ VAL-overexpressing LAD cells labeled with luciferase developed systemic metastases, emitting strong bioluminescent signals, whereas more than 5 × 10^4^ vector-control cells presented only weak systemic bioluminescent signals (Fig. 2h, i and Supplementary Fig. 3i). Indeed, micro-computed tomography imaging and histological staining revealed prominent cancerous lesions and tissue invasion/disruption in the lower-limb bones of nude mice intracardially injected with VAL-overexpressing LAD cells, whereas vector-control cells displayed far lower ability to spread to bone tissue (Fig. 2j). Consequently, nude mice intracardially injected with VAL-overexpressing LAD cells had shorter metastasis-free and overall survival than those with vector-control cells (Supplementary Fig. 3j). On the contrary, silencing VAL drastically abrogated the abilities of H1975 cells to form systemic metastases (Fig. 2h–j and Supplementary Fig. 3i, j). Furthermore, in the orthotopic implantation model, while the growth of orthotopic xenografts of vector-control A549 or H2009 cells in the lung tissue was exclusively restricted in few lesion sites, VAL-overexpressing LAD cells formed a number of different cancerous lesions in various lobes of the lungs; by contrast, silencing VAL in H1975 cells significantly suppressed intra-pulmonary metastases (Supplementary Fig. 3k–m). These both in vitro and in vivo data strongly suggest a potent pro-invasive and pro-metastatic role of VAL in LAD progression.

### VAL is important for activated AKT-induced LAD metastasis

**Fig. 3:**
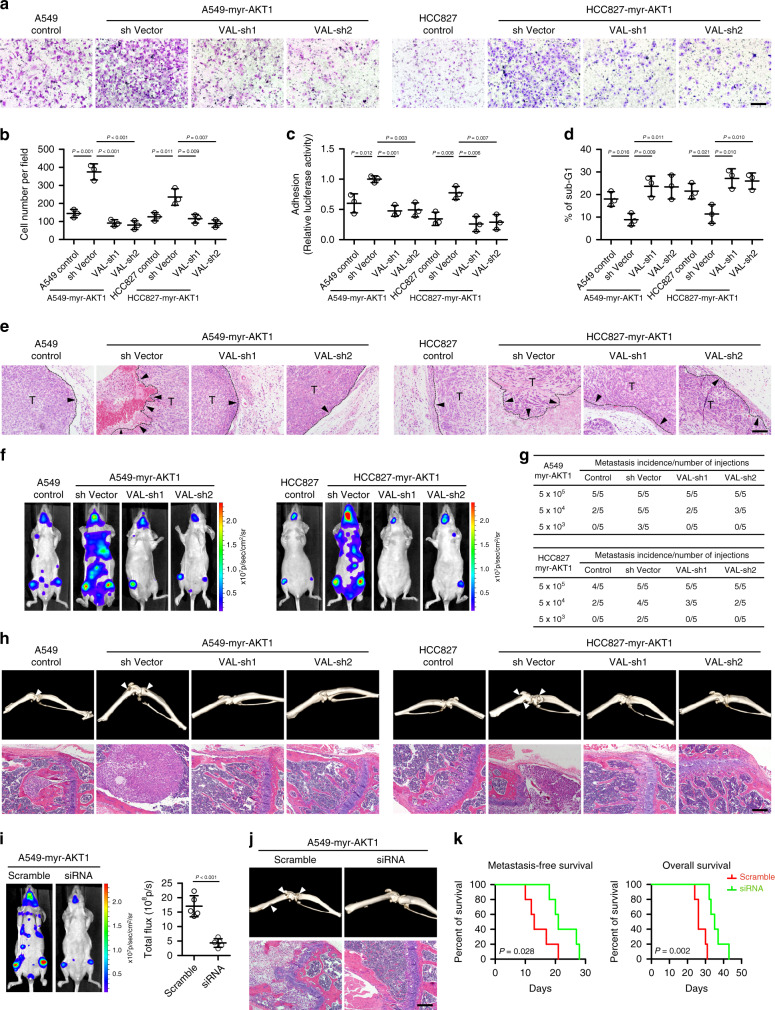
VAL is important for activated AKT-induced LAD metastasis. **a**, **b** Representative images and quantification of invading cells in five random fields of Matrigel-coated transwell assay. **c** Relative luciferase activities of myr-AKT1-overexpressing LAD cells silenced with VAL or corresponding vector-control cells adhering to the Matrigel. **d** The sub-G1 DNA contents of detached cells for myr-AKT1-overexpressing LAD cells silenced with VAL or corresponding vector-control cells are shown in the anoikis assay. **e** H&E staining of both the dermal tissue and tumor tissue in the subcutaneous tumors xenografted with the indicated cells. Black arrows mark the direction of tumor invasion with invasive front marked by dashed line. **f** Myr-AKT1-overexpressing LAD cells silenced with VAL or corresponding vector-control cells labeled with luciferase expression were injected via cardiac ventricle into nude mice (*n* = 5 per group). Representative bioluminescent images of systemic metastasis are shown. **g** Frequencies of formation of metastatic lesions in nude mice (*n* = 5 per group) intracardially injected with different cell numbers of the indicated cells. **h** Metastatic bone lesions in mice intracardially injected with the indicated cells were confirmed by micro-CT imaging and H&E staining. **i** Myr-AKT1-overexpressing LAD cells were intracardially injected into nude mice (*n* = 5 per group), followed by intravenous injection with chemosynthetic VAL siRNAs or control scramble siRNAs. Representative bioluminescence images of systemic metastasis are shown, and quantitation of bioluminescent intensities as analyzed by ROI tools. **j** The effect of intravenous injection of VAL siRNAs on the formation of metastatic bone lesions in nude mice intracardially injected with A549-myr-AKT1 cells was evaluated by micro-CT imaging and H&E staining. **k** Kaplan–Meier analysis (Log-rank test) of the effect of antagonizing VAL on the metastasis-free survival and overall survival of nude mice intracardially injected with myr-AKT1-overexpressing LAD cells. Scale bar: 100 μm (**a**, **e**, **h**, **j**). Scatter dot plots represent the means ± SD derived from three independent experiments. Statistical analyses were performed by two-way ANOVA multiple comparison analysis (**b**–**d**) and two-tailed unpaired Student’s *t* test (**i**). Source data are provided as a Source Data file.

To further illustrate the functional importance of VAL in overactivated AKT-induced LAD malignancy, myr-AKT1-overexpressing A549 and HCC827 cells silenced for VAL, and the vector-control cells, were constructed and analyzed for their invasive and metastatic abilities (Supplementary Fig. 4a). As shown in Fig. 3a–d, silencing VAL markedly reversed the promoting effect of overactivated AKT1 on cell invasion, adhesion, and anoikis resistance. Moreover, while tumor cells in each of the xenografts formed by myr-AKT1-overexpressing LAD cells generated invasive fronts and irregularly invaded into the neighboring subcutaneous tissue, nearly all the tumor xenografts formed by vector-control cells or VAL-silenced myr-AKT1-overexpressing LAD cells presented clear tissue boundary; notably, silencing VAL failed to reverse the promoting effect of AKT1 on tumor cell growth (Fig. 3e and Supplementary Fig. 4b, c). In parallel, silencing VAL drastically abrogated the abilities of myr-AKT1-overexpressing LAD cells to form systemic metastases, as evidenced by the compromised systemic bioluminescent signals, almost intact bone tissue without cancerous lesion, and prolonged metastasis-free and overall survival of nude mice intracardially injected with myr-AKT1-overexpressing LAD cells silenced for VAL (Fig. 3f–h and Supplementary Fig. 4d, e). Importantly, intravenous injection of chemosynthetic siRNAs against VAL into nude mice intracardially injected with myr-AKT1-overexpressing LAD cells greatly diminished systemic bioluminescent signals, protected bone tissues from metastatic lesions, and improved metastasis-free and overall survival (Fig. 3i–k). In the orthotopic implantation model, silencing VAL significantly suppressed intra-pulmonary metastases induced by constitutively activated AKT (Supplementary Fig. 4f, g). Taken together, these data strongly demonstrate the crucial and potent pro-metastatic role of VAL in constitutively activated AKT-driven LAD metastasis and a great therapeutic potential of targeting VAL for metastatic LAD patients.

### VAL interacts with Vimentin to inhibit Vimentin degradation

**Fig. 4:**
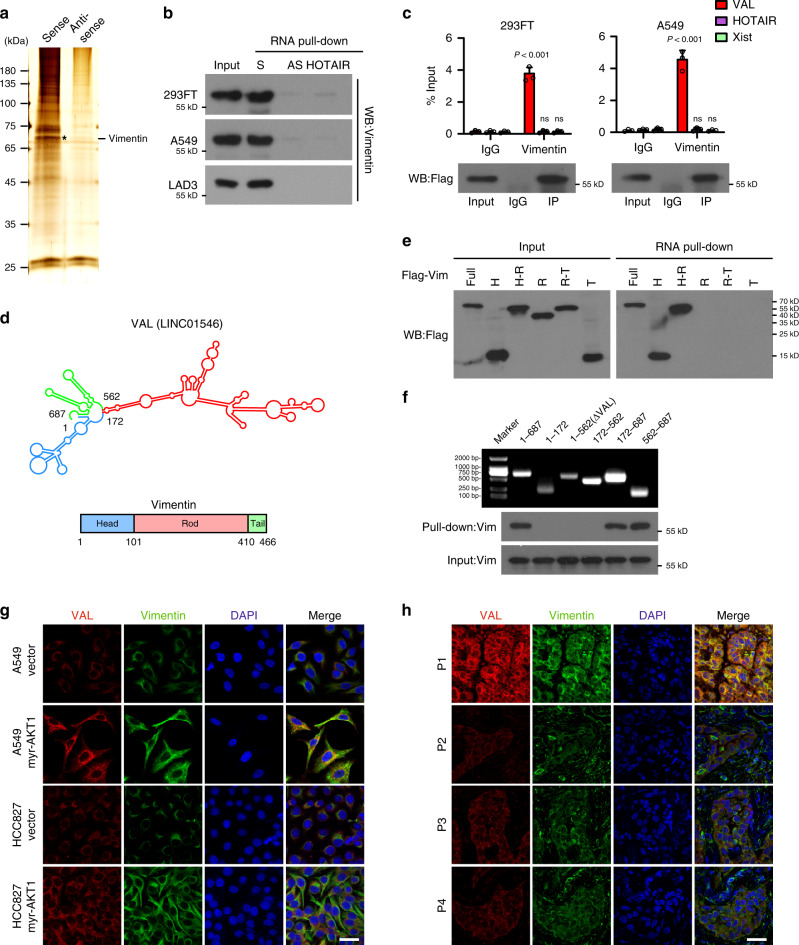
VAL interacts with Vimentin protein. **a** Proteins bound to sense or antisense RNA of VAL were separated by SDS-PAGE following endogenous RNA pull-down assay and manifested by silver staining. Distinct protein band in the gel was separately cut, dissolved, and subjected to mass spectrometry. The representative image with the silver staining of three independent endogenous RNA pull-down assays is shown. Black asterisk denotes the location of Vimentin. **b** Exogenous RNA pull-down assays validate the specific interaction between VAL and Vimentin in the indicated cells. Antisense RNA of VAL and HOTAIR were used as negative controls. Representative images of three independent reproducible experiments are shown. **c** RIP assays show the interaction of Vimentin with VAL, but not with HOTAIR or Xist. **d** Schematic diagram of the predicted secondary structure of VAL RNA and of Vimentin protein domains. **e**, **f** Exogenous RNA pull-down assays show the interaction between VAL and different truncated mutants of Vimentin or between Vimentin and different truncated mutants of VAL. Representative images of three independent reproducible experiments are shown. **g**, **h** Representative images of co-staining of VAL and Vimentin in vector-control and myr-AKT1-overexpressing LAD cells (**g**), as well as in clinical LAD specimens (**h**) with activating *PIK3CA* (E545K) mutation (P1) or with wild-type PIK3CA or AKT (P2, P3, and P4) using smFISH and immunostaining assays together (five random fields of view per slice, DAPI: 4′, 6-diamidino-2-phenylindole, scale bar: 35 μm). Scatter dot plots represent the means ± SD derived from three independent experiments. Statistical analyses were performed by two-way ANOVA multiple comparison analysis (**c**). Source data are provided as a Source Data file.

Notably, we found that several genes adjacent to the *VAL* gene locus, such as PRKX, MXRA5, and ARSF, are slightly deregulated in the TCGA LAD datasets, and that silencing VAL did not affect expression of these adjacent genes, suggesting that VAL might not function as a cis-acting gene (Supplementary Fig. 5a, b). When RNA pull-down experiments were performed using a mixture of 18 probes (Supplementary Table 2) against endogenous VAL in A549 cells overexpressing myr-AKT1 and analyzed the binding protein by using mass spectroscopy. We found that among the top-ranked putative binding proteins of VAL (Supplementary Data 2), Vimentin, which has been well-recognized as an EMT marker and whose biological importance in tumor metastasis in a non-EMT context is largely unknown, was identified and validated to interact with VAL (Fig. 4a, b and Supplementary Fig. 5c). RNA immunoprecipitation (RIP) assay further confirmed the specific interaction of Flag-tagged Vimentin with VAL (Fig. 4c). Moreover, RNA pull-down and RIP assays using various truncated constructs of both Vimentin and VAL molecules revealed that the Head domain of Vimentin proteins intensively interacted with segment 562–687 nt of VAL (Fig. 4d–f and Supplementary Fig. 5d, e). Notably, mutant VAL molecules (ΔVAL) deleted for segment 562–687 nt could not bind to Vimentin (Fig. 4f and Supplementary Fig. 5e). Interestingly, single-molecule RNA fluorescence in situ hybridization showed not only colocalization of VAL and Vimentin in the cytoplasm of LAD cells, but also upregulation of staining intensities of Vimentin proteins in VAL-overexpressing myr-AKT1 LAD cells (Fig. 4g). Similarly, human LAD specimens showed colocalization of VAL and Vimentin in the cytoplasm of lung cancer cells, and LAD specimens carrying activating *PIK3CA* (E545K) mutation presented increased staining intensities of both VAL and Vimentin as compared to those carrying wild-type *PIK3CA* or *AKT* (Fig. 4h). These data indicate that the binding of VAL to Vimentin proteins might increase Vimentin protein levels in LAD.

**Fig. 5:**
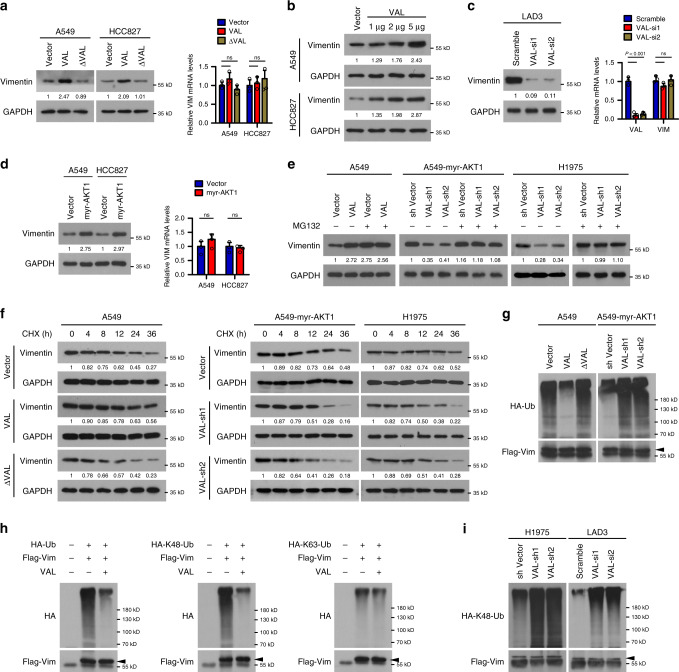
VAL inhibits Ubiquitin-mediated Vimentin degradation. **a** The effect of overexpressing Vimentin-interactive VAL or ΔVAL unable to bind Vimentin on protein and mRNA levels of Vimentin. **b** WB analysis of Vimentin expression in LAD cells transfected with various dosages of VAL expressing- or control-vector plasmids. **c** The effect of silencing VAL on the protein and mRNA levels of Vimentin in primary LAD cells LAD3. **d** The effect of overexpressing myr-AKT1 on protein and mRNA levels of Vimentin. **e** WB analysis of Vimentin expression in the indicated cells treated with or without MG132. **f** The effect of overexpressing VAL or ΔVAL, or silencing VAL on the half-life of Vimentin was evaluated in the indicated cells treated with cyclohexamide (CHX) and collected at the indicated time points. **g**–**i** The effect of overexpressing Vimentin-interactive VAL or ΔVAL, or silencing VAL on the levels of K48-, or K63-linked polyubiquitination of Vimentin was evaluated by immunoprecipitation of FLAG-tagged Vimentin in control-vector- or myr-AKT1-overexpressing A549 cells or VAL-silenced H1975 and LAD3 cells. Representative images derived from one of three independent experiments are presented. Relative protein levels were quantified by scanning densitometry and calculated as Band Intensity of Protein of Interest/Band Intensity of GAPDH (**a**–**f**). Error bars represent the means ± SD derived from three independent experiments. Statistical analyses were performed by two-tailed unpaired Student’s *t* test (**a**, **c**, **d**). n.s., not significant. Source data are provided as a Source Data file.

Indeed, overexpressing full-length VAL, but not ΔVAL that is unable to bind Vimentin, markedly increased Vimentin protein levels, whereas hardly affected the level of Vimentin mRNA and overexpression of VAL increased only the protein levels of Vimentin in a dose-dependent manner (Fig. 5a, b). In parallel, using a Tet-on inducible system in A549 cells, addition of doxycycline (Dox) induced VAL expression and markedly upregulated the protein levels, but not the mRNA levels, of Vimentin (Supplementary Fig. 5f). By contrast, silencing VAL in cultured primary lung cancer cells (LAD3) originated from a stage III LAD patient’s primary lung tumor, in which VAL was also highly expressed and able to bind Vimentin protein (Fig. 4b), significantly suppressed the protein level rather than the mRNA level of Vimentin (Fig. 5c). Moreover, Vimentin protein levels, but not its mRNA levels, were also significantly increased in myr-AKT1-overexpressing LAD cells and silencing VAL reversed the inducing effect of constitutively activated AKT on Vimentin protein levels (Fig. 5d, e). We then used proteasome inhibitor MG132 to determine whether VAL affects Vimentin degradation. As shown in Fig. 5e, overexpressing or silencing VAL hardly changed Vimentin protein levels when proteasome-mediated protein degradation was blocked by MG132 treatment in vector-control or myr-AKT1-overexpressing A549 cells or H1975 cells. Moreover, overexpressing full-length VAL, but not ΔVAL, extended the protein half-life of Vimentin, while silencing VAL significantly accelerated degradation of Vimentin proteins and reversed the promoting effect of activated AKT on Vimentin protein stability (Fig. 5f). Furthermore, overexpression of only Vimentin-interactive VAL caused remarkable suppression of K48-linked, but not K63-linked polyubiquitination of Vimentin proteins in LAD cells; consistently, polyubiquitination of Vimentin proteins was markedly diminished in myr-AKT1-overexpressing LAD cells and silencing VAL greatly promoted K48-linked Vimentin polyubiquitination in H1975 and LAD3 cells and reversed the inhibitory effect of activated AKT on Vimentin polyubiquitination (Fig. 5g–i). In addition, insulin or FGF2 treatment faintly decreased polyubiquitination of Vimentin protein and thus marginally increased Vimentin protein levels (Supplementary Fig. 5g, h), indicating an importance of VAL induced by fully activated AKT/STAT3 signaling in the process of Vimentin upregulation. Taken together, these data suggest that the binding of VAL to Vimentin proteins abrogates polyubiquitination-mediated degradation of Vimentin proteins and thus increases Vimentin protein levels.

### VAL abrogates the Vimentin–Trim16 interaction

**Fig. 6:**
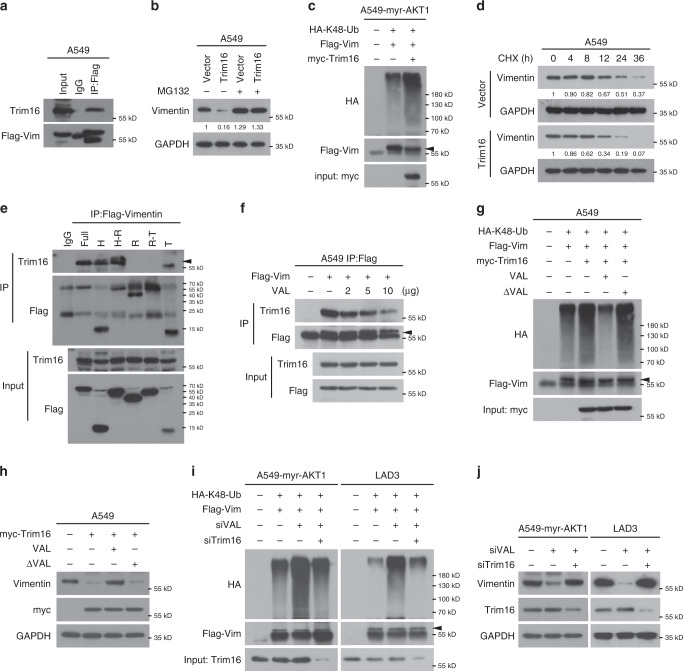
VAL abrogates Trim16-induced degradation of Vimentin. **a** Co-IP assay following immunoprecipitation of Flag-tagged Vimentin shows the interaction of Vimentin with Trim16. **b** WB analysis of Vimentin expression in vector-control or Trim16-overexpressing A549 cells treated with or without MG132. **c** The effect of overexpressing Trim16 on the levels of K48-linked polyubiquitination of Vimentin was evaluated by immunoprecipitation of FLAG-tagged Vimentin in myr-AKT1-overexpressing A549 cells. **d** The half-life of Vimentin proteins were evaluated in vector-control or Trim16-overexpressing A549 cells treated with CHX and collected at the indicated time points. **e** Interaction between Trim16 and different truncated mutants of Flag-tagged Vimentin was evaluated by co-IP assays in 293FT cells. **f** The effect of overexpressing VAL in a dose-dependent manner on the interaction between Trim16 and Flag-tagged Vimentin as evaluated by immunoprecipitation of Flag. **g** The effect of overexpressing Trim16 on the levels of K48-linked polyubiquitination of Vimentin in the presence of overexpressed VAL or of ΔVAL was evaluated by immunoprecipitation of FLAG-tagged Vimentin in A549 cells. **h** WB analysis of Vimentin levels in A549 cells with the indicated treatments. **i** The effect of silencing VAL or together with silencing Trim16 on the levels of K48-linked polyubiquitination of Vimentin in A549-myr-AKT1 or LAD3 cells. **j** WB analysis of Vimentin levels in A549-myr-AKT1 or LAD3 cells silenced with VAL or together with Trim16 knockdown. Representative images derived from one of three independent experiments are presented. Relative protein levels were quantified by scanning densitometry and calculated as Band Intensity of Protein of Interest/Band Intensity of GAPDH (**b**, **d**). Source data are provided as a Source Data file.

In light that Trim16, an E3 ligase, was previously reported as a binding partner and inhibitor of Vimentin^29^, we then investigated whether Trim16 causes Vimentin degradation and VAL could abrogate this process in LAD cells. As shown in Fig. 6a–d, Trim16 indeed could bind to Vimentin, decreased Vimentin protein levels in a manner dependent of proteasome-mediated degradation, caused K48-linked polyubiquitination of Vimentin and thus shortened the half-life of Vimentin proteins to <12 h in LAD cells, suggesting that Trim16 is a putative E3 ligase for Vimentin, at least in LAD cells. Notably, we further found that Trim16 bound to the Head domain of Vimentin (Fig. 6e), which also was the region of Vimentin binding to VAL as aforementioned, indicating that VAL might competitively bind to Vimentin and consequently abrogate the interaction between Trim16 and Vimentin. Indeed, addition of VAL decreased the binding of Trim16 to Vimentin in a dose-dependent manner in LAD cells (Fig. 6f). Moreover, overexpression of only Vimentin-interactive VAL could largely abrogate Trim16-induced K48-linked polyubiquitination and thus decrease of Vimentin protein levels (Fig. 6g, h). In parallel, silencing VAL failed to cause Vimentin polyubiquitination or decrease Vimentin protein levels when Trim16 was pre-silenced in myr-AKT1-overexpressing A549 cells, H1975 or LAD3 cells (Fig. 6i, j and Supplementary Fig. 5i, j). Taken together, these data suggest that VAL competitively binds to Vimentin and thus abrogates the interaction of Vimentin with its E3 ligase Trim16, resulting in disruption of Trim16-dependent degradation of Vimentin proteins.

### Blocking Vimentin degradation is crucial for LAD metastasis

**Fig. 7:**
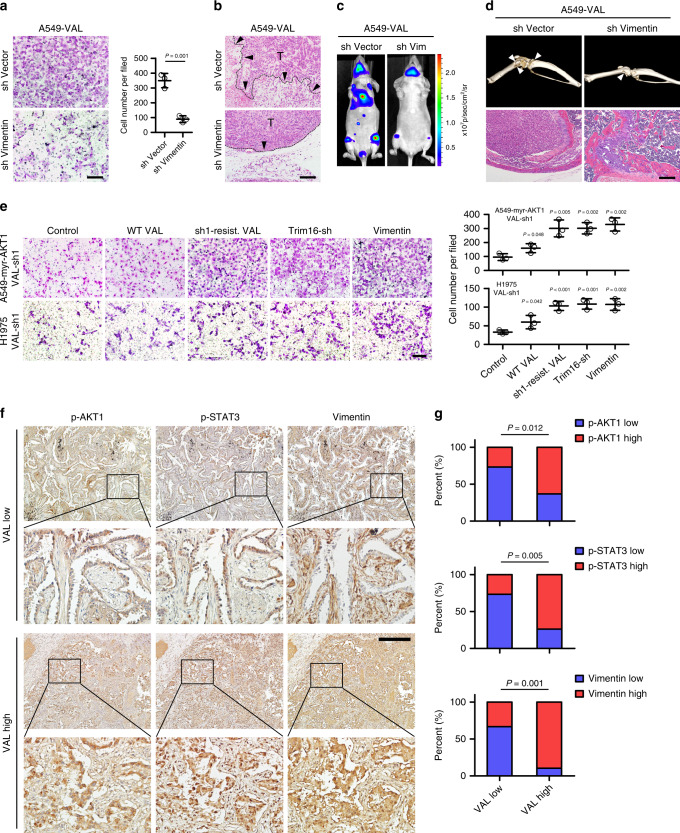
Blocking Vimentin degradation is crucial for LAD metastasis. **a** Representative images and quantification of invading cells in five random fields of Matrigel-coated transwell assay. **b** Representative images of H&E staining display a clear boundary and irregular invasive front between the dermal tissue and tumor tissue in the subcutaneous tumors (*n* = 5 per group), respectively, xenografted with the indicated cells. Black arrows mark the direction of tumor invasion with invasive front marked by dashed line. **c** Representative bioluminescent images of systemic metastasis in nude mice (*n* = 5 per group) intracardially injected with VAL-overexpressing A549 cells silenced with Vimentin or corresponding vector-control cells. **d** Representative images of metastatic bone lesions in nude mice (*n* = 5 per group) intracardially injected with A549-VAL cells silenced with Vimentin or control scramble were evaluated by micro-CT imaging and H&E staining. **e** The effect of overexpressing Vimentin, silencing Trim16, or restoring an shRNA-resistant mutant VAL or wild-type VAL on cell invasion in A549-myr-AKT1 or H1975 cells pre-silenced for VAL. **f**, **g** Expression of VAL is associated with activated AKT (Ser-473 phosphorylated AKT1), activated STAT3 (Ser-727 phosphorylated STAT3) and Vimentin levels in LAD tissue. Two representative cases are shown. Scale bar: 100 μm (**a**, **b**, **d**, **e**), 200 μm (**f**). Scatter dot plots represent the means ± SD. Statistical analyses were performed by two-way ANOVA multiple comparison analysis (**e**, **g**) and two-tailed unpaired Student’s *t* test (**a**). Source data are provided as a Source Data file.

Subsequently, we asked whether the Trim16-Vimentin pathway was important for the potent pro-metastatic role of VAL in LAD cells. As shown in Fig. 7a and Supplementary Fig. 6a–c, silencing Vimentin greatly reversed the promoting effect of VAL on cell invasion, adhesion, and anoikis resistance. Consistently, tumor cells in the xenografts formed by VAL-overexpressing LAD cells pre-silenced for Vimentin hardly generated invasive front or invaded into the neighboring subcutaneous tissue; meanwhile, silencing Vimentin did not affect tumor growth (Fig. 7b and Supplementary Fig. 6d, e). Similarly, VAL-overexpressing LAD cells pre-silenced for Vimentin exhibited bioluminescent signals as weak as vector-control LAD cells, in contrast to the prominent systemic metastatic signals resulting from VAL-overexpressing LAD cells when these LAD cells were separately injected intracardially; as a consequence, nude mice intracardially injected with VAL-overexpressing LAD cells pre-silenced for Vimentin had significantly prolonged metastasis-free and overall survival as compared to those bearing systemic metastases of vector-control LAD cells overexpressing VAL (Fig. 7c, d and Supplementary Fig. 6f, g). Notably, ΔVAL unable to bind Vimentin hardly potentiated the abilities of LAD cells to adhere to or invade through the Matrigel, or resist anoikis (Supplementary Fig. 6h–j). Moreover, silencing VAL failed to reverse the promoting effects of overactivated AKT on cell adhesion, invasion, and anoikis resistance when Trim16 was pre-silenced or Vimentin was re-introduced in LAD cells (Fig. 7e and Supplementary Fig. 6k, l). Similar to the consequence of Vimentin overexpression or Trim16 depletion, restoring the shRNA-resistant mutant of VAL could fully reverse the inhibitory effects of silencing VAL on cell invasion, adhesion, and anoikis resistance, whereas overexpression of wild-type VAL, which mildly restored VAL levels in VAL-silenced LAD cells, slightly rescued the invasive and metastatic abilities of VAL-silenced LAD cells (Fig. 7e and Supplementary Fig. 6k–n), suggesting that VAL indeed works in trans by stabilizing Vimentin to promote LAD metastasis. Importantly, positive correlations between high VAL expression and high levels of AKT1 and STAT3 activities, and Vimentin expression were consistently found in our collected cohort. Specifically, 63.16%, 73.68%, 89.47% of 19 cases of LAD tissue expressing high VAL showed high levels of AKT and STAT3 phosphorylation and Vimentin expression, respectively; in parallel, 73.33%, 73.33%, 66.67% of 15 cases of LAD tissue expressing low VAL showed low levels of AKT and STAT3 phosphorylation and Vimentin expression, respectively (Fig. 7f, g), further supporting the tight clinical relevance of the AKT-STAT3-VAL-Vimentin axis in LAD progression. Taken together, our data strongly suggest that overactivated AKT-induced VAL promotes LAD metastasis through blocking Trim16-dependent proteasomal degradation of Vimentin.

## Discussion

It has been widely well-recognized that aberrant AKT activity is the central hub of numerous signaling pathways driven by activation of oncogenes or loss of tumor suppressors, such as EGFR and other receptor tyrosine kinases, Ras, PI3K, BRAF, AKT itself, and natural AKT inhibitor PTEN, and plays pivotal role during tumor development and progression^30,31^. In fact, the majority of NSCLC tumors, especially the LAD subtype, harbor distinct genetic mutations in one or some of these driver oncogenes causing overactivated AKT signaling^32^. It is highly notable that while clinical therapy with molecular drugs targeting these driver oncogenes may exhibit therapeutic efficiency at first due to dramatic suppression of AKT signaling, drug resistance usually rapidly develops, especially in metastatic LAD patients, which in many cases is relevant to AKT re-overactivation^33^. It appears that simultaneously targeting AKT or its crucial downstream effectors might be more effective therapeutic approaches. Unfortunately, to date none of AKT inhibitors is approved for anti-cancer therapy in clinic, including those with high specificity and low adverse effects and toxicity as shown in preclinical tests^33^. In parallel, although many protein effectors downstream to the AKT signaling significantly contribute to cancer metastasis, few of them can be used as effective therapeutic targets in NSCLC. For example, targeted therapy against mTOR, a vital substrate of AKT, has been approved for the treatment of breast, renal, and pancreatic cancers but not lung cancer, and the suboptimal responsiveness in tested NSCLC patients remains to be an unresolved issue^34^. Therefore, there is an urgent need to identify other unknown effectors of aberrant AKT signaling as clinically applicable therapeutic targets, particularly for metastatic NSCLC patients.

Similar to protein-coding regulators, recently mounting studies have revealed that lncRNAs are also importantly involved in tumor development and progression and have great potentials to be clinically applied in the treatment and diagnosis of cancer^19,35^. On the backdrop that active AKT-induced lncRNAs crucial for the malignant behaviors of tumor cells had not been well characterized, we conducted this current study and identified VAL (LINC01546), which is directly transcribed by high STAT3 activity induced by overactivated AKT, as a potent pro-metastatic non-coding molecule. Specifically, overexpressing VAL strikingly promoted, whereas silencing VAL suppressed local invasion and intra-pulmonary and distant metastasis, and greatly reversed overactivated AKT-induced tumor invasion and metastasis. Impressively, systemic administration of siRNA antagonist of VAL greatly diminished systemic bioluminescent signals, protected bone tissues from metastatic lesions and improved metastasis-free and overall survival of nude mice intracardially injected with myr-AKT1-overexpressing LAD cells. Moreover, high VAL level correlates with disease progression and poor outcome of clinical LAD patients. These data suggest both diagnostic and therapeutic potentials of VAL for metastatic LAD patients, in particular for those driven by AKT overactivation.

Since EMT is crucial, although not indispensable, for initiating the process of tumor metastasis cascade, Vimentin has gained much attention as an essential marker for EMT^8,36,37^. However, in the scenario of EMT-related metastasis, although the reversal phenomenon of EMT, namely, mesenchymal-epithelial transition is thought to occur during colocalization of tumor cells in the secondary metastatic foci, Vimentin can often be detected in tumor cells in these metastases^38,39^. In addition, increased Vimentin expression can be widely found in various epithelial cancers, including lung cancer, even with epithelial phenotypes^40–42^. These studies suggest that the function of Vimentin in cancer metastasis may be more than just a marker of EMT^43–45^. In such a context, our current study finds that stabilization of Vimentin proteins can be crucial for AKT/VAL-induced cancer cell adhesion, invasion, and survival, consequently resulting in local and distant metastasis of LAD cells, supporting the notion that abnormal levels of Vimentin may play pivotal biological roles at multiple levels in the complex process of LAD metastasis. This finding may facilitate a better understanding of Vimentin-related tumor metastasis and also suggests modulation of Vimentin expression as a potential therapeutic approach, especially for metastatic LAD patients.

Despite the biological significance and increased expression of Vimentin proteins (but not mRNA levels) found in LAD tissue, the regulatory mechanism underlying aberrant Vimentin expression has not been investigated in non-EMT scenario. Several lines of evidence suggest that Vimentin expression is regulated by posttranslational modifications^46,47^. For example, skeletal muscle cells use a degradation-initiating complex mainly consisting of ubiquilin2 (UBQLN2) and myotubularin-1 (MTM1) to recognize misfolded intermediate filament proteins such as Vimentin and Desmin for degradation, which ensures cytoskeletal integrity to avoid proteotoxic aggregate formation^48^. In ovarian cancer cells, Trim56 has been found to be the ubiquitin ligase for Vimentin and suppress tumor invasion and migration^49^. Our data demonstrate that Trim16 is the E3 ligase for Vimentin in LAD cells, and that the binding of VAL to Vimentin competitively abrogates Trim16-induced polyubiquitination and thus degradation of Vimentin. Interestingly, we also found that VAL is hardly detectable in some mesenchymal cells such as HFL1 and MRC5 (Supplementary Table 1), where both Trim16 and Vimentin are normally expressed, and overexpressing VAL or Trim16 in mesenchymal cells failed to change protein levels of Vimentin, whereas overexpressing VAL readily increased, and overexpressing Trim16 decreased Vimentin expression in non-cancerous and cancerous epithelial cells (Supplementary Fig. 1 only for Reviewer), suggesting that the VAL-Vimentin-Trim16 interaction model is a unique system for epithelial cells. Notably, several previous reports suggest that Trim16 acts as a tumor suppressor based on its decreased expression in multiple types of cancers and its inhibitory effects on tumor cell proliferation, survival, invasion, and migration^29,50–54^. However, moderate to strong staining of Trim16 proteins and even a slightly increased mRNA level of Trim16 could be found in LAD tissue from various public datasets^55^. Therefore, our study not only illustrates a mechanism underlying abrogation of Vimentin degradation during active AKT-driven LAD progression but also provides a foundation for developing Vimentin stability as a diagnostic marker or a therapeutic target for anti-cancer strategy.

## Methods

### Cell culture

LAD cell lines, including A549, HCC827, H1650, H596, H1975, H1299, H292, H2009, and H2030 and non-cancerous HEK293FT, HFL1, and MRC5 cells were purchased from the American Type Culture Collection (ATCC; Manassas, VA, USA) and maintained in Dulbecco’s modified Eagle’s medium (DMEM for LAD cell lines and HEK293FT cells; GIBCO, Carlsbad, CA, USA), Improved Minimum Essential Medium (IMEM for MRC5 cell; GIBCO) or Ham’s F-12K Medium (for HFL1 cells; GIBCO) supplemented with 10% fetal bovine serum (Corning, Tewksbury, MA, USA) and 1% penicillin/streptomycin (penicillin 100 U/ml and streptomycin 10 μg/ml) (GIBCO). LAD3 cells were isolated from the tumor biopsy collected from a stage III LAD patient, and cultured in Defined Keratinocyte SFM (GIBCO) supplemented with L-glutamine, EGF (20 ng/ml), basic-FGF (10 ng/ml), 2% B27, penicillin/streptomycin, and amphotericin B (0.25 mg/ml)^56,57^. The Beas2B immortalized human bronchial epithelial cell line obtained from ATCC was cultured in Bronchial Epithelial Cell Growth Medium as instructed by the supplier (Lonza, Walkersville, MD, USA). All cell lines were authenticated by short tandem repeat DNA profiling at Medicine Laboratory of Forensic Medicine Department of Sun Yat-Sen University (Guangzhou, China), and were verified to be mycoplasma-free.

### Clinical specimens and ethical approval

All clinical tissue specimens used in this study were obtained from and histopathologically diagnosed at Sun Yat-Sen Memorial Hospital. LAD tissue and paired adjacent non-cancerous lung specimens collected during surgery were frozen in liquid nitrogen and stored at −40 °C until further use. Adjacent non-cancerous specimens were obtained from a standard distance (3 cm) from the tumor margin in resected tissues of LAD patients. For the use of these clinical materials for research purposes, prior patients’ consents and approval from the Institutional Research Ethics Committee of Sun Yat-sen University were obtained. The study is compliant with all relevant ethical regulations involving human participants.

### TCGA dataset analysis and sequencing data deposition

RNAseq data for lncRNA expression of 38 pairs of LAD tissues and paired adjacent non-cancerous lung tissues, and 495 cases of LAD tissues, as well as their corresponding clinical information, were mined from The Cancer Genome Atlas lung cancer datasets [https://cancergenome.nih.gov/]. In addition, total RNAs from vector-control and myr-AKT1-overexpressing A549 or HCC827 cells were enriched for polyadenylated RNAs using the Poly(A) RNA Selection Module and collected for strand-specific sequencing analysis by Berry Genomics (Beijing, China). Sequenced reads were trimmed for adapter sequence, and masked for low-complexity or low-quality sequence, then mapped to HG38 whole genome using Bowtie2 version 2.3.5. Reads Per Kilobase of exon per Megabase of library size values were calculated using HTseq version 0.11.2. Sequencing data have been deposited in GEO database with accession number GSE136904.

### Statistical analysis

All statistical analyses except the sequencing data were performed using the PASW Statistics 18 version 18.0.0 (SPSS Inc., Chicago, IL, USA) software package and GraphPad Prism 8 version 8.3.0 (GraphPad Software, San Diego, CA, USA). Survival curves were analyzed by the Kaplan–Meier method and compared by the log-rank test. Comparisons between two groups were performed using Student’s *t* test (two-tailed), while analyses comparing multiple treatments with a control group were performed using two-way ANOVA with post hoc Dunnett’s multiple comparisons test. All error bars represent the mean ± SD derived from three independent experiments. In all cases, *p* < 0.05 was considered to be statistically significant.

### Study approval

All experimental procedures and use of LAD donors’ samples were approved by the SYSU Institutional Animal Care and Use Committee, and Research Ethics Committee. Donors provided prior written informed consent.

### Reporting summary

Further information on research design is available in the Nature Research Reporting Summary linked to this article.

## Supplementary information

Supplementary Information

Peer Review File

Description of Additional Supplementary Data Files

Supplementary Dataset 1

Supplementary Dataset 2

Reporting Summary

## Data Availability

The TCGA Lung Adenocarcinoma (TCGA-LUAD) sequencing data used in this study are available in a public repository from the GDC Data Portal (https://portal.gdc.cancer.gov/). The RNA-sequencing data that support the findings of this study have been deposited in GEO with the accession code GSE136904. All other data supporting the findings of this study are available from the corresponding author upon reasonable request. Source data are provided with this paper.

## Source data

Source Data
