# Supplementary material for: AKT-induced lncRNA VAL promotes EMT-independent metastasis through diminishing Trim16-dependent Vimentin degradation
Source: Nat Commun. 2020 Oct 12;11:5127. doi: 10.1038/s41467-020-18929-0 (PMC7550350; doi:10.1038/s41467-020-18929-0)
Supplement: Supplementary file 1 — Supplementary Information [file 41467_2020_18929_MOESM1_ESM.pdf]

Supplementary Information

AKT-induced lncRNA VAL promotes EMT-independent metastasis through diminishing Trim16-dependent Vimentin degradation

Tian, et al.

Supplementary figures

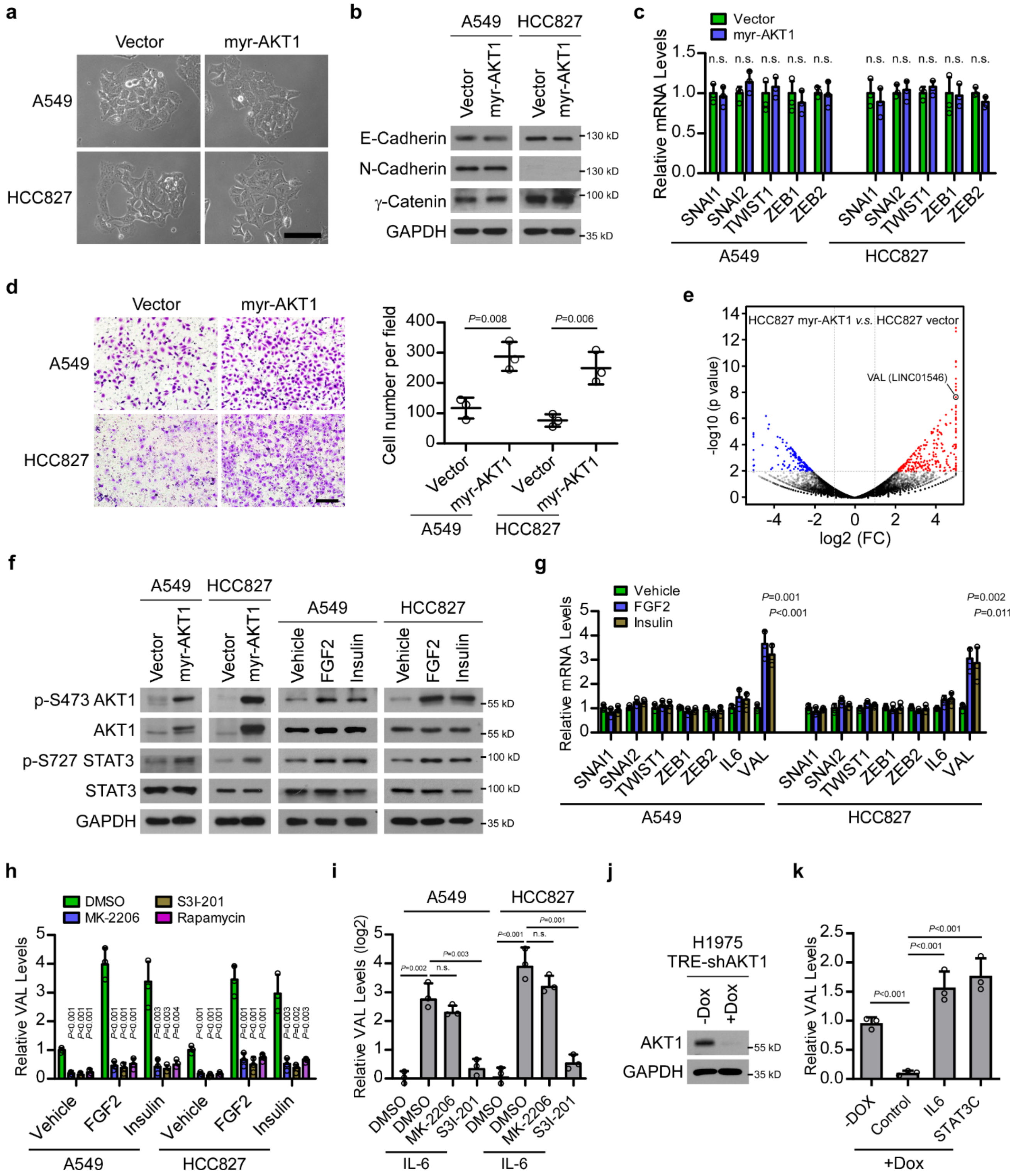

Supplementary Figure 1. AKT/STAT3 signaling activation induces VAL expression.

a Representative bright-field morphology images in 5 random fields of vector-control or myr-AKT1-overexpressing A549 and HCC827 cells. Scale bar: 30 μm. **b, c** WB and qRT-PCR analysis shows that

EMT-related cellular markers or transcriptional factors are not altered by constitutively active AKT. **d** Representative images and quantification of invading cells in 5 random fields of Matrigel-coated transwell assay. Scale bar: 100 µm. **e** Volcano plot shows the significantly up-regulated (red) and down-regulated (blue) lncRNAs in HCC827-myr-AKT1 cells as compared to HCC827-vector cells based on RNA sequencing. **f** WB analysis of activation of AKT and STAT3 by constitutively active AKT or by transient stimulation with recombinant FGF2 or insulin. The representative images of three independent reproducible experiments are shown. **g** QRT-PCR analysis of the effect of FGF2 or insulin stimulation on expression of the indicated genes in A549 or HCC827 cells. **h** The effect of treatment of AKT, STAT3 or mTOR inhibitors or together with FGF2 or insulin stimulation on VAL expression in the indicated cells. **i** QRT-PCR analysis shows the reversing effect of AKT or STAT3 inhibitors on IL6-induced VAL expression in A549 and HCC827 cells. **j** WB analysis validated the effect of Doxycycline (Dox)-induced AKT1 depletion in H1975 cells transduced with the pLVX-TRE3G-shAKT1 Tet-On inducible system. **k** The effect of Dox-induced AKT1 depletion or together with stimulation with recombinant IL6 protein or overexpression of constitutively active STAT3 (STAT3C) on VAL expression. Error bars represent the means ± SD derived from three independent experiments. Statistical analyses were performed by two-way ANOVA multiple comparison analysis (**i, k**) and two-tailed unpaired Student's *t* test (**c, d, g, h**). n.s., not significant. Source data are provided as a Source data file.

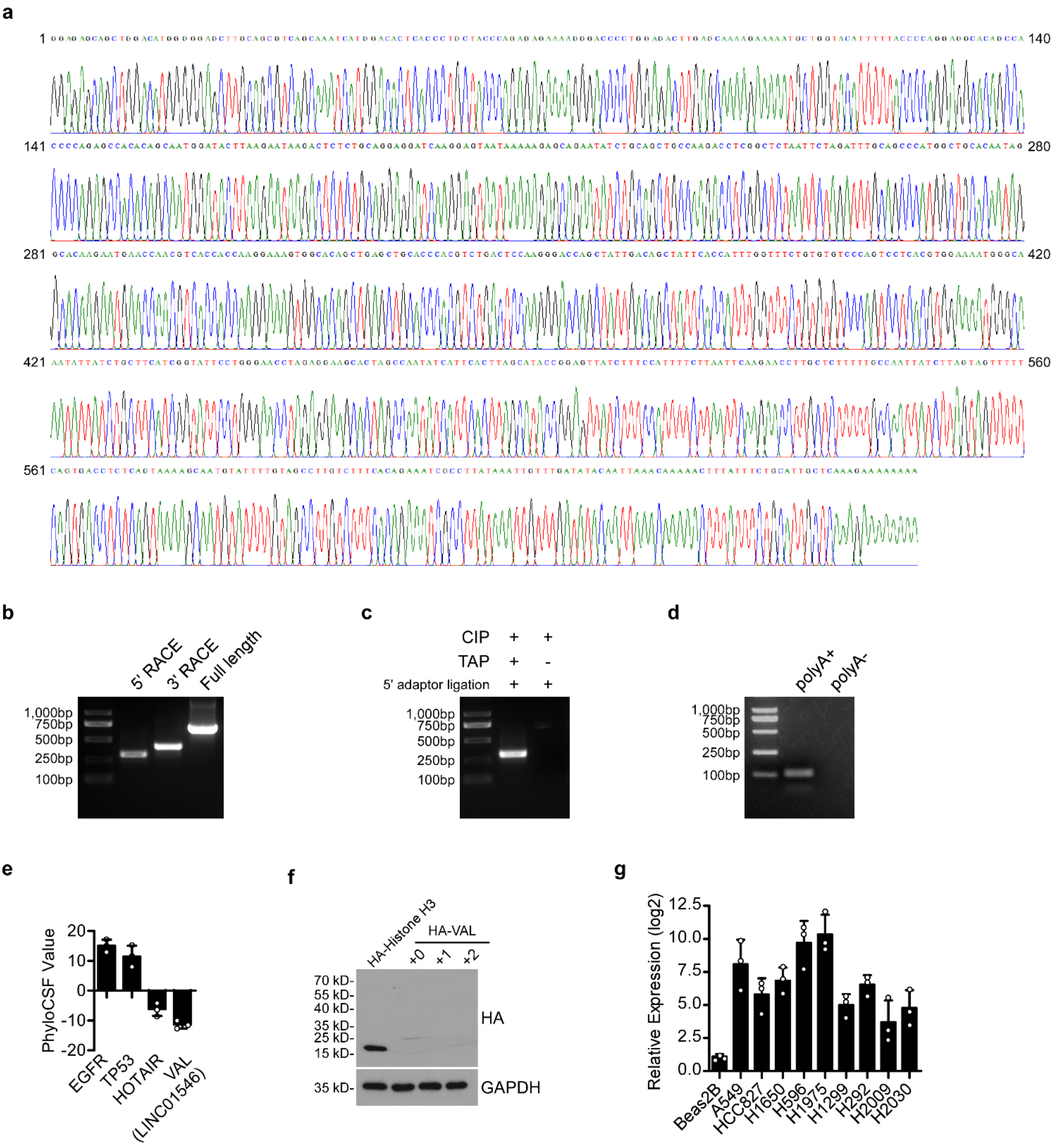

Supplementary Figure 2. VAL is a long non-coding RNA and highly expressed in LAD cells.

a Sequences of full-length VAL (LINC01546). **b** Representative image of PCR products of VAL from the 5'-RACE and 3'-RACE assays. **c** Total RNAs deprived of 5' and 3' phosphorylation ends by calf intestine alkaline phosphatase (CIP) were treated with or without tobacco acid pyrophosphatase (TAP), which disrupts the cap structure, followed by ligation with an RNA adapter oligonucleotide. RT-PCR detection of VAL suggests that VAL RNA has the cap structure. **d** RT-PCR analysis of VAL in

polyA+ RNA fraction and polyA- RNA fraction in A549 cells. **e** PhyloCSF analysis shows the codon substitution frequency (CSF) scores of VAL, HOTAIR, EGFR and TP53. Error bars represent the means ± SD of the PhyloCSF scores of three truncated RNA sequences of indicated mRNAs. **f** WB analysis of the coding potency of VAL when full length of VAL was cloned into pcDNA3.4 with a transcription initiating codon ATG and a C-terminal HA tag. Anti-HA antibody was used to probe transcribed proteins. Histone H3 with HA tag served as a positive control. **g** QRT-PCR analysis of the relative VAL levels in a panel of LAD cell lines as compared to the immortalized human bronchial epithelial cell line Beas2B. The representative images of three independent reproducible experiments are shown (**b-d, f**). Error bars represent the means ± SD derived from three independent experiments. Source data are provided as a Source data file.

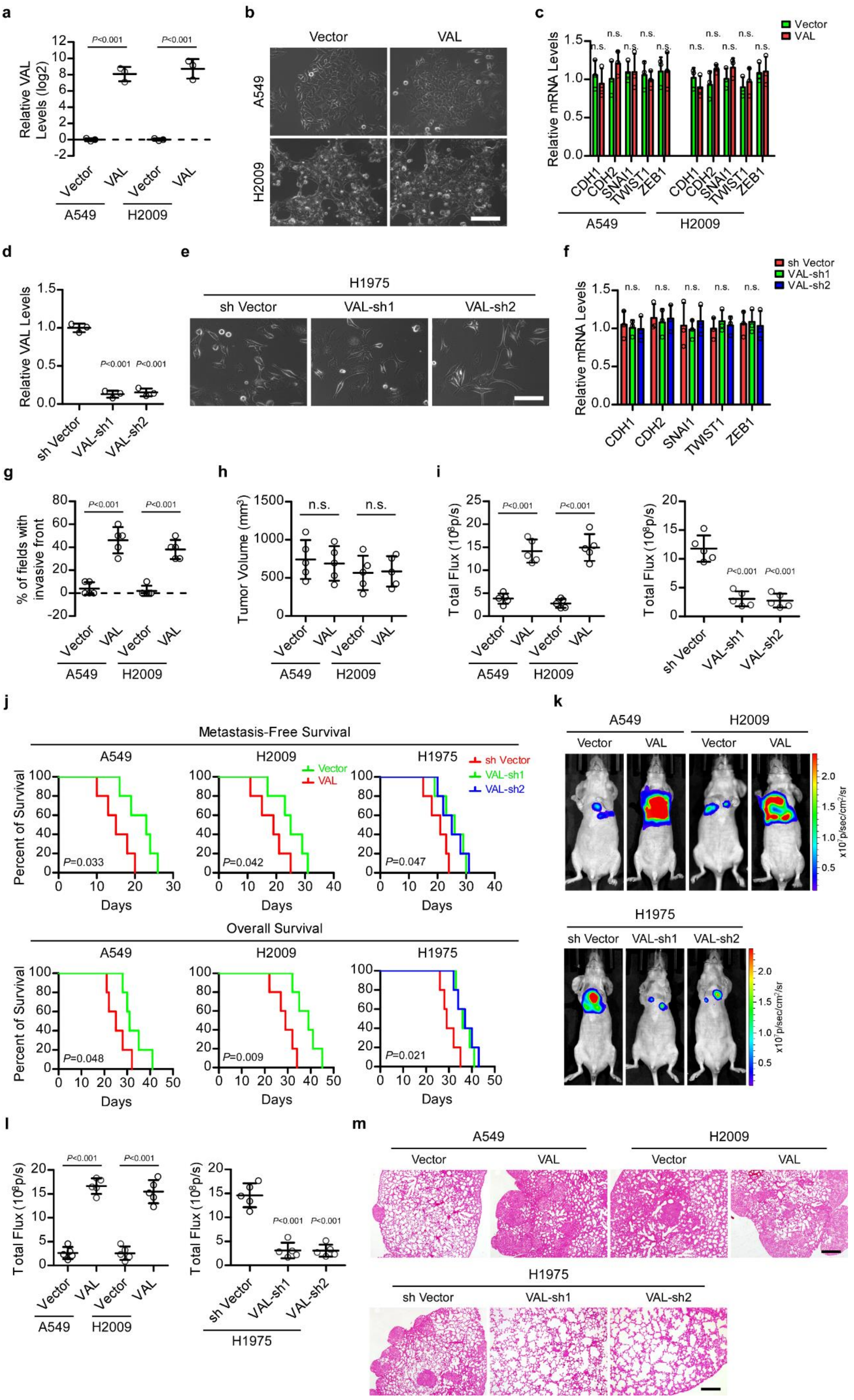
a
Relative VAL Levels (log2)
P<0.001
P<0.001
Vector
VAL
Vector
VAL
A549
H2009
b
Vector
VAL
A549
H2009
c
Vector
VAL
Relative mRNA Levels
n.s.
CDH1
CDH2
SNAI1
TWIST1
ZEB1
A549
H2009
d
Relative VAL Levels
P<0.001
P<0.001
sh Vector
VAL-sh1
VAL-sh2
e
H1975
sh Vector
VAL-sh1
VAL-sh2
f
sh Vector
VAL-sh1
VAL-sh2
Relative mRNA Levels
n.s.
CDH1
CDH2
SNAI1
TWIST1
ZEB1
g
% of fields with invasive front
P<0.001
P<0.001
Vector
VAL
Vector
VAL
A549
H2009
h
Tumor Volume (mm3)
n.s.
n.s.
Vector
VAL
Vector
VAL
A549
H2009
i
Total Flux (10^8p/s)
P<0.001
P<0.001
Vector
VAL
Vector
VAL
A549
H2009
Total Flux (10^8p/s)
P<0.001
P<0.001
sh Vector
VAL-sh1
VAL-sh2
j
Metastasis-Free Survival
A549
H2009
H1975
Vector
VAL
sh Vector
VAL-sh1
VAL-sh2
Percent of Survival
P=0.033
P=0.042
P=0.047
Days
Overall Survival
A549
H2009
H1975
Percent of Survival
P=0.048
P=0.009
P=0.021
Days
k
A549
H2009
Vector
VAL
Vector
VAL
x10^7p/sec/cm²/sr
H1975
sh Vector
VAL-sh1
VAL-sh2
x10^7p/sec/cm²/sr
l
Total Flux (10^8p/s)
P<0.001
P<0.001
Vector
VAL
Vector
VAL
A549
H2009
Total Flux (10^8p/s)
P<0.001
P<0.001
sh Vector
VAL-sh1
VAL-sh2
H1975
m
A549
H2009
Vector
VAL
Vector
VAL
H1975
sh Vector
VAL-sh1
VAL-sh2

Supplementary Figure 3. VAL promotes local invasion and intra-pulmonary and distant metastasis without inducing EMT.

a QRT-PCR analysis of VAL levels in vector-control and VAL-overexpressing LAD cells. **b** Representative bright-field morphology images in 5 random fields of vector-control or VAL-overexpressing A549 and HCC827 cells. **c** QRT-PCR analysis shows that EMT-related cellular markers or transcriptional factors are not altered by VAL overexpression. **d-f** The effect of silencing VAL on cellular morphology or expression of EMT-related cellular markers or transcriptional factors in H1975 cells. qRT-PCR analysis of VAL levels in vector-control and VAL-silencing LAD cells. The representative images in 5 random fields of indicated cells are shown (**e**). **g** Scatter dot plots present the frequency of tumor xenografts showing invasion of tumor cells into the neighboring subcutaneous tissue in ten microscopic fields under 200× magnification, as well as the number of microscopic fields with invasive fronts between the tumor area and the neighboring subcutaneous tissue per slice of each tumor xenograft of the indicated cells. **h** Comparison of tumor volumes of subcutaneous xenografts inoculated with vector-control or VAL-overexpressing LAD cells. **i** Quantitation of bioluminescent intensities as analyzed by ROI tools in nude mice intracardially injected with VAL-overexpressed or -silenced LAD cells or corresponding vector-control cells. **j** Kaplan-Meier analysis (Log-rank test) of the metastasis-free survival and overall survival of mice intracardially injected with the indicated cells. **k-m** VAL-overexpressed or -silenced LAD cells or corresponding vector-control cells labeled with luciferase expression were orthotopically implanted into the lung of nude mice (n = 5 per group). Representative bioluminescent images of pulmonary metastasis and quantitation of bioluminescent intensities analyzed by ROI tools are shown. Lung metastases were histologically confirmed by H&E staining. Scale bar: 30 μm (**b, e**), 100 μm (**m**). Error bars represent the means ± SD derived from three independent experiments. Statistical analyses were performed by two-tailed unpaired Student's *t* test (**a, c, d, f-i, l**). n.s., not significant. Source data are provided as a Source data file.

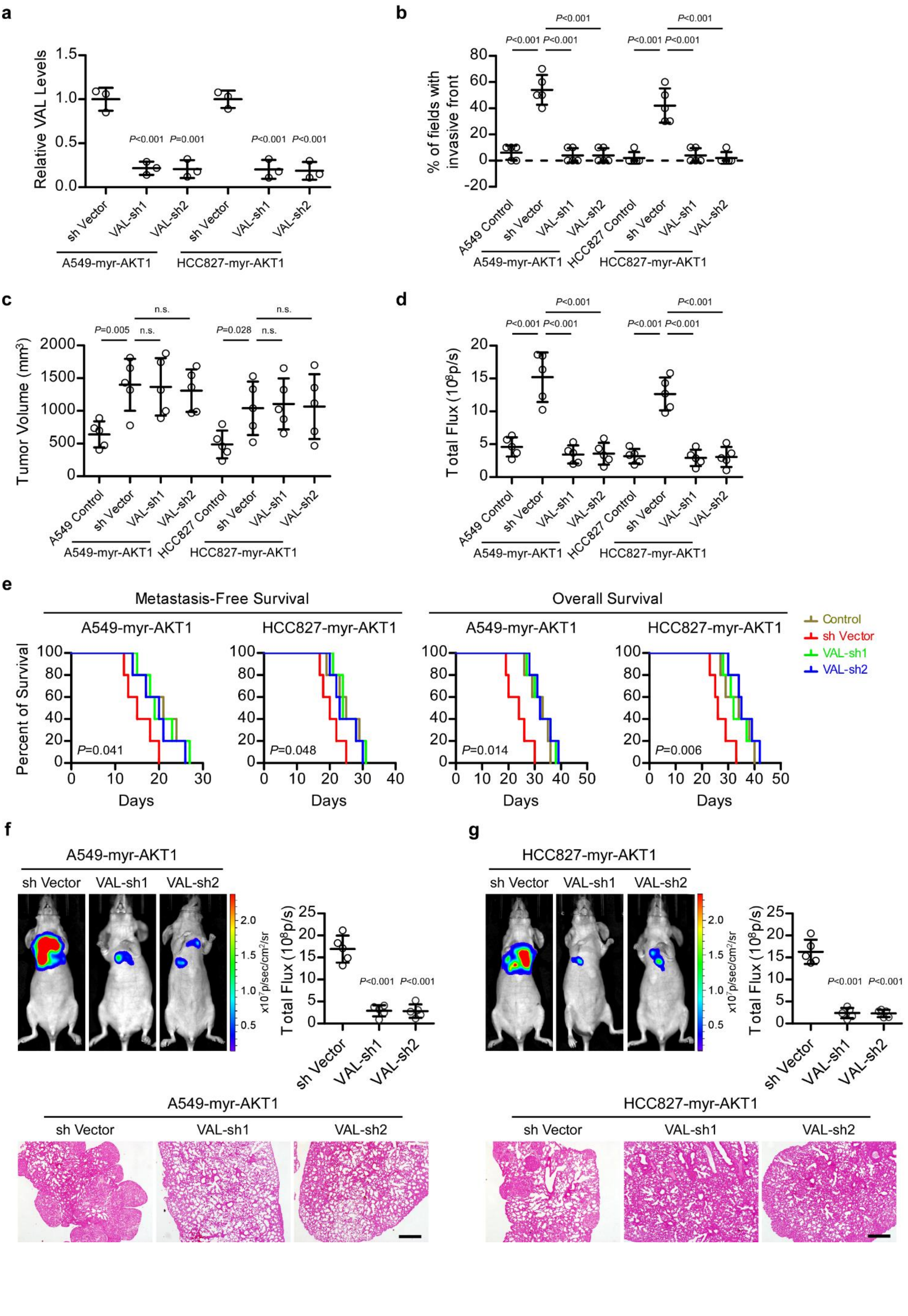

a
Relative VAL Levels
P<0.001
P=0.001
P<0.001
P<0.001
sh Vector
VAL-sh1
VAL-sh2
A549-myr-AKT1
HCC827-myr-AKT1
b
% of fields with invasive front
P<0.001
A549 Control
HCC827 Control
c
Tumor Volume (mm3)
P=0.005
P=0.028
n.s.
d
Total Flux (10^8p/s)
e
Metastasis-Free Survival
Overall Survival
Control
Percent of Survival
Days
P=0.041
P=0.048
P=0.014
P=0.006
f
g
x10^7p/sec/cm^2/sr

Supplementary Figure 4. VAL is important for activated AKT-induced LAD metastasis.

a QRT-PCR analysis of VAL levels in myr-AKT1-overexpressing LAD cells silenced with VAL or control scramble. **b** Scatter dot plots present the frequency of tumor xenografts showing invasion of tumor cells into the neighboring subcutaneous tissue in ten microscopic fields under 200× magnification, as well as the number of microscopic fields with invasive fronts between the tumor area and the neighboring subcutaneous tissue per slice of each tumor xenograft of the indicated cells. **c** Comparison of tumor volumes of subcutaneous xenografts inoculated with myr-AKT1-overexpressing LAD cells silenced with VAL or control scramble. **d** Quantitation of bioluminescent intensities analyzed by ROI tools in nude mice intracardially injected with myr-AKT1-overexpressing LAD cells silenced with VAL or control scramble. **e** Kaplan-Meier analysis (Log-rank test) of the metastasis-free survival and overall survival of nude mice intracardially injected with the indicated cells. **f, g** myr-AKT1-overexpressing LAD cells silenced with VAL or control scramble and labeled with luciferase expression were orthotopically implanted into the lung of nude mice (n = 5 per group). Representative bioluminescent images of pulmonary metastasis, quantitation of bioluminescent intensities analyzed by ROI tools and H&E staining of lung tissue are shown. Scale bar: 100 µm. Scatter dot plot represent the means ± SD. Statistical analyses were performed by two-way ANOVA multiple comparison analysis (**b-d**) and two-tailed unpaired Student's *t* test (**a, f, g**). n.s., not significant. Source data are provided as a Source data file.

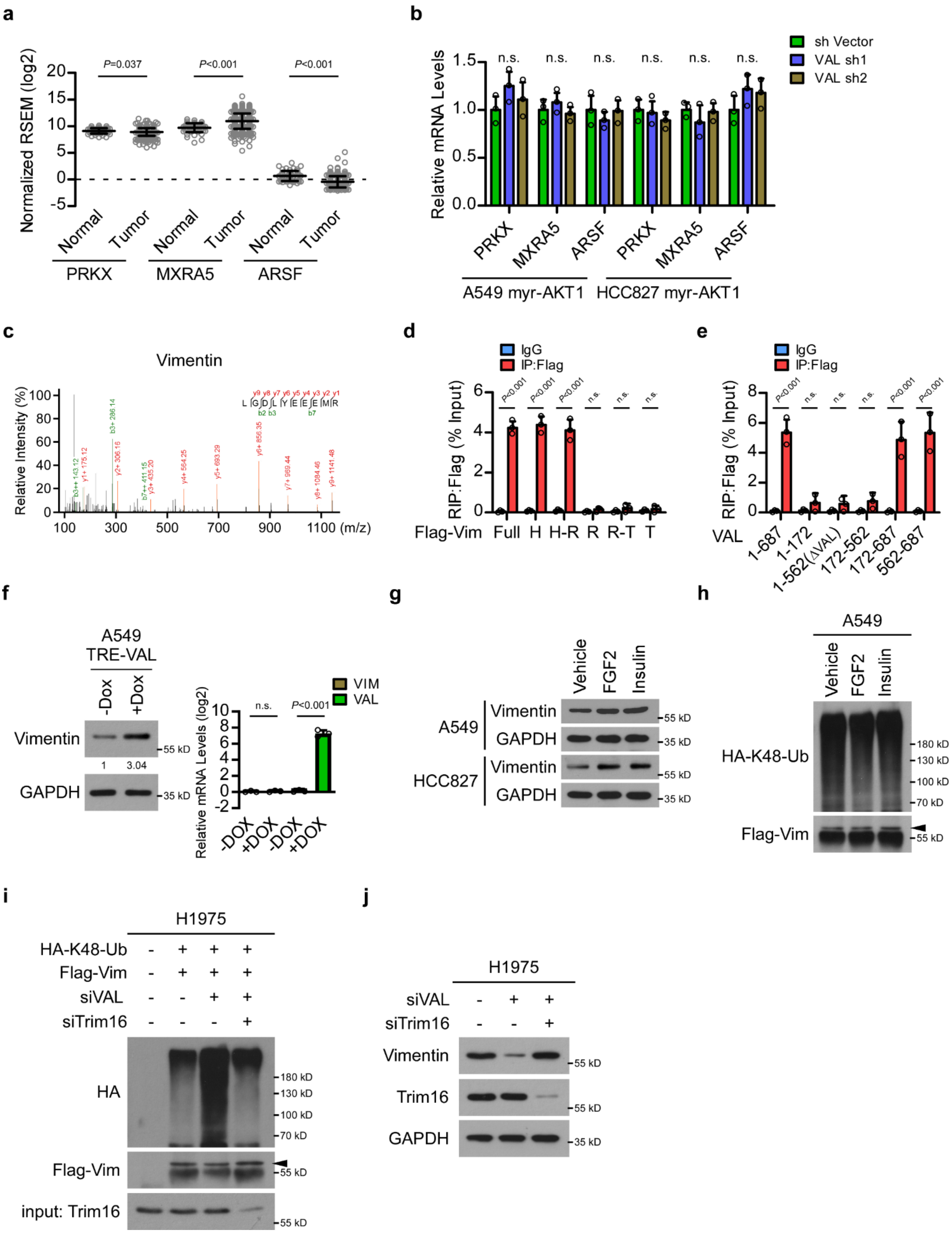

Supplementary Figure 5. VAL interacts with Vimentin protein and abrogates Trim16-induced polyubiquitination and degradation of Vimentin.

a Scatter dot plots show the expression levels of genes adjacent to the *VAL* gene locus, including PRKX, MXRA5 and ARSF in tumor and adjacent normal tissues of the TCGA LUAD datasets. **b** QRT-PCR analysis shows that silencing VAL barely alters expression of PRKX, MXRA5 or ARSF. **c** Mass spectrum plot of representative peptides of Vimentin protein identified in specific bands in the gel of WB analysis. **d, e** RIP assays show the interaction between truncated mutants of Flag-tagged Vimentin and full-length VAL or between full-length Flag-tagged Vimentin and truncated mutants of VAL. **f** WB and qRT-PCR analysis the effect of VAL upregulation induced by Dox addition using the pLVX-TRE3G-VAL Tet-On inducible expression system on protein and mRNA levels of Vimentin. **g, h** The effect of FGF2 or insulin stimulation on expression of Vimentin proteins or K48-linked polyubiquitination of Vimentin. **i** The effect of silencing VAL or together with silencing Trim16 on the levels of K48-linked polyubiquitination of Vimentin in H1975 cells. **j** WB analysis of Vimentin levels in H1975 cells silenced with VAL or together with Trim16 knockdown. The representative images of three independent reproducible experiments are shown (**g-j**). Error bars represent the means ± SD of three independent experiments. Statistical analyses were performed by two-tailed unpaired Student's *t* test (**a, b, d-f**). n.s., not significant. Source data are provided as a Source data file.

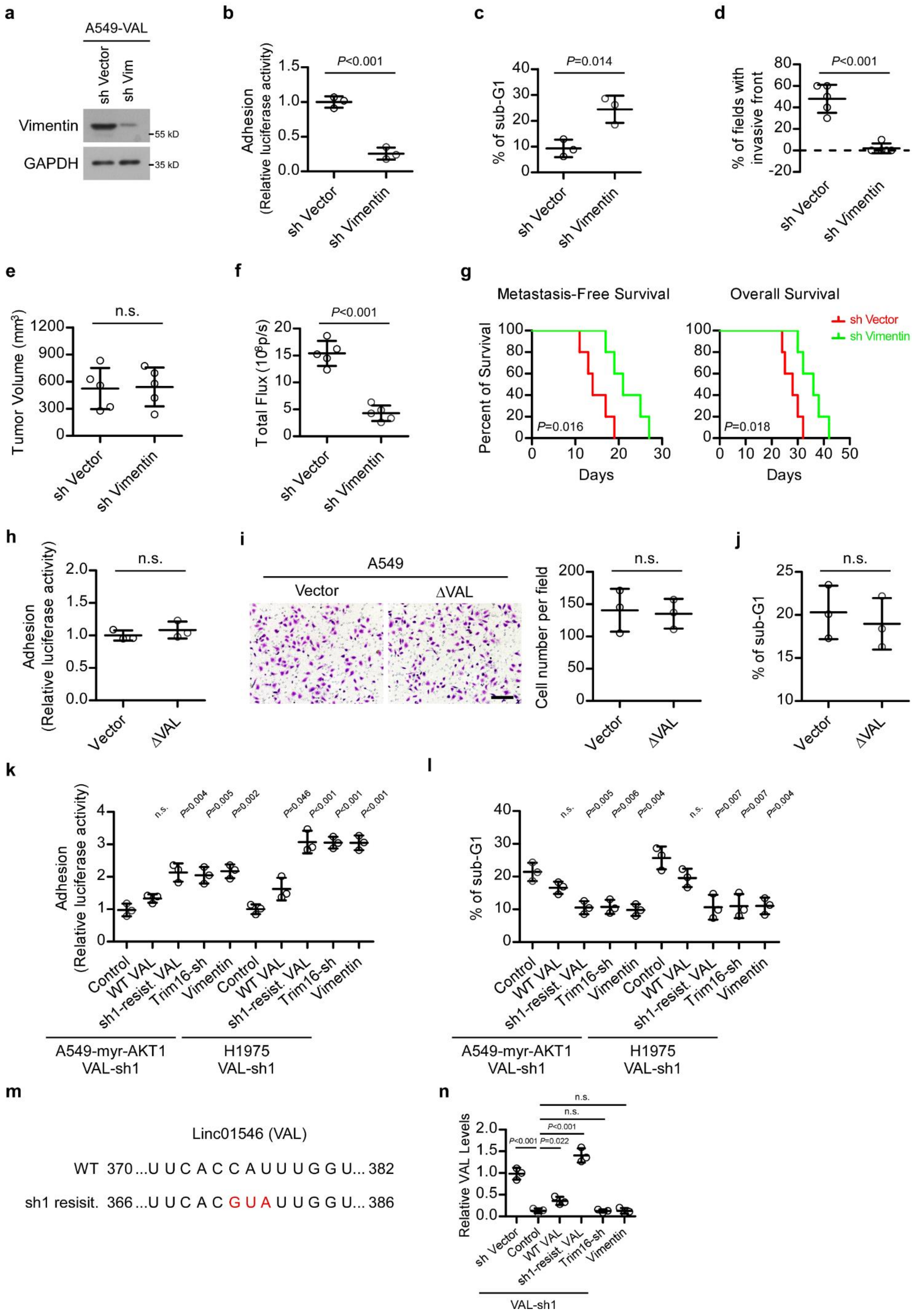
a
A549-VAL
sh Vector
sh Vim
Vimentin
GAPDH
55 kD
35 kD
b
Adhesion (Relative luciferase activity)
P<0.001
sh Vector
sh Vimentin
c
% of sub-G1
P=0.014
sh Vector
sh Vimentin
d
% of fields with invasive front
P<0.001
sh Vector
sh Vimentin
e
Tumor Volume (mm3)
n.s.
sh Vector
sh Vimentin
f
Total Flux (108p/s)
P<0.001
sh Vector
sh Vimentin
g
Metastasis-Free Survival
Overall Survival
sh Vector
sh Vimentin
Percent of Survival
P=0.016
P=0.018
Days
h
Adhesion (Relative luciferase activity)
n.s.
Vector
ΔVAL
i
A549
Vector
ΔVAL
Cell number per field
n.s.
j
% of sub-G1
n.s.
Vector
ΔVAL
k
Adhesion (Relative luciferase activity)
n.s.
P=0.004
P=0.005
P=0.002
P=0.046
P<0.001
P<0.001
P<0.001
Control
WT VAL
sh1-resist. VAL
Trim16-sh
Vimentin
A549-myr-AKT1 VAL-sh1
H1975 VAL-sh1
l
% of sub-G1
n.s.
P=0.005
P=0.006
P=0.004
n.s.
P=0.007
P=0.007
P=0.004
Control
WT VAL
sh1-resist. VAL
Trim16-sh
Vimentin
A549-myr-AKT1 VAL-sh1
H1975 VAL-sh1
m
Linc01546 (VAL)
WT 370...U U C A C C A U U U G G U...382
sh1 resisit. 366...U U C A C G U A U U G G U...386
n
Relative VAL Levels
P<0.001
P=0.022
P<0.001
n.s.
n.s.
sh Vector
Control
WT VAL
sh1-resist. VAL
Trim16-sh
Vimentin
VAL-sh1

Supplementary Figure 6. Blocking Vimentin degradation is crucial for LAD metastasis.

a WB analysis of Vimentin levels in VAL-overexpressing A549 cells silenced with Vimentin or control scramble. The representative images of three independent reproducible experiments are shown. **b** Relative luciferase activities of VAL-overexpressing A549 cells silenced with Vimentin or corresponding vector-control cells adhering to the Matrigel. **c** The sub-G1 DNA contents of detached cells for VAL-overexpressing A549 cells silenced with Vimentin or corresponding vector-control cells are shown in the anoikis assay. **d** Scatter dot plots present the frequency of tumor xenografts showing invasion of tumor cells into the neighboring subcutaneous tissue in ten microscopic fields under 200× magnification, as well as the number of microscopic fields with invasive fronts between the tumor area and the neighboring subcutaneous tissue per slice of each tumor xenograft of the indicated cells. **e** Comparison of tumor volumes of subcutaneous xenografts inoculated with A549-VAL cells silenced with Vimentin or control scramble. **f** Quantitation of bioluminescent intensities of systemic metastasis in nude mice (n = 5 per group) intracardially injected with VAL-overexpressing A549 cells silenced with Vimentin or corresponding vector-control cells. **g** Kaplan-Meier analysis (Log-rank test) of the metastasis-free survival and overall survival of mice intracardially injected with VAL-overexpressing A549 cells silenced with Vimentin or corresponding vector-control cells. **h-j** The effect of overexpressing ΔVAL on cell adherence, invasion and anoikis resistance in A549 cells. Scale bar: 100 µm. **k, l** The effect of silencing Trim16, or restoring Vimentin, full-length wild-type VAL or shRNA1-resistant mutated VAL on cell adherence and anoikis resistance in A549-myr-AKT1 and H1975 cells silenced with VAL. **m** Partial sequences of full-length wild-type VAL and shRNA1-resistant mutated VAL are shown. **n** QRT-PCR analysis of the relative VAL levels in VAL-silenced H1975 cells with the indicated treatments. Scatter dot plot represent the means ± SD of three independent experiments. Statistical analyses were performed by two-way ANOVA multiple comparison analysis (**k, l, n**) and two-tailed unpaired Student's *t* test (**b-f, h-j**). n.s., not significant. Source data are provided as a Source data file.

Supplementary Table 1. Absolute quantification of VAL expression

Sample	Ct Value	Copies per cell
A549 Vector	28.59	~41
A549 myr-AKT1	16.30	~937
A549 VAL	19.78	~386
HCC827 Vector	29.32	~34
HCC827 myr-AKT1	17.08	~768
H2009 Vector	31.22	~21
H2009 VAL	22.65	~186
H1975 shVector	23.07	~167
H1975 VAL-sh1	32.54	~15
H1975 VAL-sh2	30.86	~23
Beas2B	35.01	~8
Normal lung 1	40.46	~2
Normal lung 2	35.54	~7
Lung adenocarcinoma Phase I #1	25.37	~93
Lung adenocarcinoma Phase I #2	27.5	~54
Lung adenocarcinoma Phase II #1	24.41	~119
Lung adenocarcinoma Phase II #2	22.61	~188
Lung adenocarcinoma Phase III #1	22.25	~206
Lung adenocarcinoma Phase III #2	21.6	~243
Lung adenocarcinoma Phase III #3*	16.42	~908
Lung adenocarcinoma Phase IV #1	18.21	~576
Lung adenocarcinoma Phase IV #2	20.47	~324
HFL1	>45	~0
MRC5	>45	~0

X: Log_2(molecular number); Y: Ct Value; Equation: Y = -2.723*X + 43.48

*Lung adenocarcinoma Phase III #3 is harboring *PIK3CA* E545K mutation.

Supplementary Table 2. Probes for smFISH and RNA pull-down assay

Probe #	Probe (5'-> 3')	Probe Position
1	tgagtgtccatgatttgctg	33nt-52nt
2	cttttctctctgggtagcag	55nt-74nt
3	cctcctggggtaaaaatgta	111nt-130nt
4	tcttaagtatccattgctgt	151nt-170nt
5	tctgctctttttattactcc	196nt-215nt
6	tagaattagagccgaggtct	232nt-251nt
7	tcattcttgtgcctattgtg	271nt-290nt
8	tttccttggtggtgacgttg	293nt-312nt
9	cttggagtcagacgtgggtg	330nt-349nt
10	aatagctgtcaatagctggt	352nt-371nt
11	gaggactgggacacacagaa	383nt-402nt
12	taatatttgcccattttcca	406nt-425nt
13	tcccaggaataccgatgaag	430nt-449nt
14	atgatattggctagtgcttc	458nt-477nt
15	ataactccggtatgctaagt	480nt-499nt
16	ggcaaaaagagcaaggttct	520nt-539nt
17	tgcttttactgagaggtcac	561nt-580nt
18	ggcgatttctgtgaaagaca	597nt-616nt

Supplementary Methods

Plasmids, virus production and transfection. Full-length, truncated or shRNA-resistant (mutation of three nucleotides essential for prefect pairing to a distinct shRNA sequence) VAL (LINC01546) was cloned into pSin-EF2-puro retroviral vector (Addgene, Cambridge, MA, USA) and Flag-Vimentin, Myc-Trim16 and HA-Ubiquitin were separately cloned into pCDNA3.4 TOPO vector (Invitrogen, Carlsbad, CA, USA). For depletion of VAL, Vimentin, or Trim16, human shRNA sequences against each of them were respectively cloned into the pSuper-retro-neo or pSuper-retro-puro vectors (OligoEngine, Seattle, WA, USA). Approximately 500bp DNA fragments containing the putative predicted STAT3 binding sites with wild-type or mutated sequences in the promoter/enhancer region of *LINC01546* were cloned upstream to the luciferase gene in a pGL3-Enhancer vector (Promega, Madison, WI, USA). Constitutively active STAT3C form containing A662C and N664C mutation was obtained from Addgene Inc (Cambridge, MA, USA). All siRNA oligonucleotides were purchased from Ribo (Guangzhou, China). For construction of stable cell lines overexpressing VAL or knocked down for VAL, pSin-VAL plasmid was co-transfected with pMD2G and psPAX2 packaging plasmids, and plasmid carrying distinct VAL shRNAs was co-transfected with PIK packaging plasmid in 293FT cells using a standard calcium phosphate transfection method; subsequently, cell culture supernatants of 293FT cells were collected to infect LAD cells for 24 h in the presence of polybrene (8 μg/ml), followed by selection with puromycin (1 μg/ml) or G418 (200 μg/ml) for 10-14 days. To generate Dox-inducible cell lines, cell lines were infected simultaneously with lentivirus encoding Tet-On 3G (packaged from pLVX-Tet3G) and lentivirus encoding TRE3G-myr-AKT1, TRE3G-shAKT1 or TRE3G-VAL (packaged from pLVX-TRE3G-IRES), then selected with hygromycin (200 μg/ml) and puromycin (1 μg/ml) for 2 weeks. Doxycycline hyclate (D9891; Sigma, St. Louis, MO, USA) was dissolved in ddH2O (2 mg/ml) and added to culture medium at final concentration of 2 μg/ml in order to induce overexpression or silencing of the abovementioned genes. Transfection of plasmids

or RNA oligonucleotides were performed using Lipofectamine 3000 reagent (Invitrogen) for luciferase reporter assays and molecular assays.

5’ and 3’ rapid amplification of cDNA ends (RACE) assays. 5’- and 3’- RACE assays were performed to reveal the transcriptional initiation and termination sites of VAL using SMARTer RACE 5’/3’ Kit (Takara, Tokyo, Japan) as instructed. Briefly, total RNAs were extracted from A549 and HCC827 cells using the Trizol reagent and treated with DNase I (Invitrogen) for first-strand cDNA synthesis, followed by 5’-RACE and 3’-RACE PCRs using multiple primers. PCR fragments were purified and cloned into the pMD19-T vector (Takara) and the insert with correct sequences as analyzed by sequencing was then subcloned into the expression vector plasmids.

Antibodies. Primary antibodies used for WB analysis were: anti-Vimentin (Proteintech, 60330-1-Ig, 1:2000), anti-AKT1 (Proteintech, 10176-2-AP, 1:800), anti-phosphor AKT1 (S473) (Proteintech, 66444-1-Ig, 1:800), anti-phosphor STAT3 (S727) (Cell Signaling Technology, #34911, 1:400), anti-STAT3 (Cell Signaling, #9139, 1:800), anti-E-Cadherin (Cell Signaling, #3195, 1:400), anti-N-Cadherin (Cell Signaling, #13116, 1:400), anti-γ-Catenin (Abcam, ab184919, 1:800), anti-Trim16 (Santa Cruz, sc-398851, 1:800), anti-FLAG (Sigma-Aldrich, F1804, 1:2000), anti-HA (Sigma-Aldrich, H3663, 1:2000), anti-MYC (Proteintech, 16286-1-AP, 1:2000) and Normal Mouse IgG for immunoprecipitation (Sigma-Aldrich, 12-371). Blotted membranes were stripped and re-blotted with anti-GAPDH (Proteintech, 10494-1-AP, 1:2000) used as loading controls.

RNA extraction and quantitative reverse transcription-PCR (qRT-PCR). Total RNAs from cultured cells or surgically resected fresh LAD tissues were isolated using TRIzol (Invitrogen) according to the manufacturer’s instructions. The first-strand cDNA was generated by reverse transcription using MMLV transcriptase (Promega) and random primers. Real-time qRT-PCR was performed on a CFX96 real-time PCR detection system (Bio-Rad, Richmond, CA, USA). Gene expression was assessed based on the threshold cycle (Ct), and relative expression levels were calculated as $2^{-[(\text{Ct of mRNA}) - (\text{Ct of GAPDH})]}$

after normalization to GAPDH expression.

Absolute quantification of VAL copy number. Serial dilutions of purified DNA template of VAL were used for qPCR to generate a standard curve. The copy number of the diluted DNA templates were calculated by DNA/RNA Copy Number Calculator from the following website (http://endmemo.com/bio/dnacopynum.php). To measure the VAL copy per cell, total RNAs extracted from 1×10^6 cells were reversely transcribed into cDNAs, and aliquots of cDNAs from 2,000 cells were further used for qPCR. The copy number of VAL in different cells were quantitated from the standard curve.

Cell adhesion, invasion and anoikis assays. For cell invasion assays, indicated cells (4×10^4) were plated on the top side of Transwell chambers (Corning) pre-coated with matrigel (BD Biosciences, San Jose, CA, USA) and incubated at 37°C for 24-36 hours, followed by removal of cells inside the upper chamber with cotton swabs. Cells invading to the bottom side of the membrane were fixed, stained, photographed and quantified in 5 random 200× magnification fields. For adhesion assays, indicated cells (1×10^5) labeled with luciferase expression were added to Matrigel-coated 96 well plates, and allowed to adhere at 37°C for 1.5 h. After wash of the plates twice with cold PBS, lysates of adherent cells were collected and subjected for measurement of luciferase activity using Dual-Luciferase® Reporter Assay System (Promega). For anoikis assays, polyHEMA-coated dishes were prepared by applying 2 ml polyHEMA solution (20 mg/ml polyhydroxyethylmethacrylate in ethanol; Sigma-Aldrich, Hamburg, Germany) onto 60mm dishes and air drying in a tissue culture hood. After wash with PBS for three times, indicated cells (1×10^6) were seeded into these polyHEMA-coated dishes for 48-hour culture and then collected, fixed, stained with propidium iodide, and analyzed using EPICS XL-MCL flow cytometer with EXPO32 ADC software version 1.2 (Beckman Coulter, Brea, CA, USA) for DNA content analysis.

RNA *in situ* hybridization. Staining of VAL in formalin-fixed paraffin-embedded tissues were

performed by RNA *in situ* hybridization using RNAscope 2.5 HD Detection kit from Advanced Cell Diagnostics (ACD bio, 322300) according to manufacturer's instructions. RNAscope probe Hs-LINC01546 (ACD bio, 468101) was designed by ACD for hybridization with LINC01546 and signals from hybridized probes were detected by DAB (3, 3'-diaminobenzidine). The staining scores were determined based on both the intensity and proportion of positive cells in 10 random fields. Scores representing the proportion of positively stained tumor cells in sections was graded as follows: 0, no positive cells; 1, <10%; 2, 10%-50%; and 3, >50%. The staining intensity was recorded as 0 (no staining), 1 (week staining), 2 (moderate staining), and 3 (strong staining). The staining index (SI) was calculated as follows: SI=staining intensity × proportion of positively stained cells, resulting in scores of 0, 1, 2, 3, 4, 6, or 9. The SI scores were compared between LADs and corresponding adjacent non-cancerous lung specimens by paired *t*-test.

Single-molecule RNA fluorescence *in situ* hybridization (smFISH). Staining of VAL in cultured cells was performed by smFISH according to the Sanjay Tyagi lab's procedures [1]. Short probes labeled with Alexa Fluor 647 in 3' ends were designed in an online program (https://www.biosearchtech.com/support/tools/design-software/stellaris-probe-designer) and synthesized by Invitrogen. Cells seeded on cover-slips in 24-well plates were briefly rinsed with PBS, fixed with 3.7% formaldehyde (pH 7.4) for 10 min at room temperature, permeabilized in 1 ml 70% ethanol for at least 1 h at 4°C and washed in hybridization buffer (100 mg/mL dextran sulfate and 10% formamide in 2× SSC) for 5 min. Subsequently, hybridization was carried out using anti-VAL oligonucleotide probe sets (125 nM in 50 µl hybridization buffer) for at least 4 h at 37°C in a moist chamber, followed by wash of the cells for 30 min at 37°C in wash buffer (10% formamide in 2× SSC). After the hybridization, cells on cover-slips were counterstained with 4', 6-diamidino-2-phenylindole (DAPI), and representative images were obtained with a LSM810 confocal microscope using ZEN 2012 software version 8.1 (Carl Zeiss, Oberkochen, Germany).

Co-immunoprecipitation (Co-IP) and RNA immunoprecipitation (RIP). Indicated cells were washed in PBS and lysed with cell lysis buffer supplemented with 0.2% protease inhibitor Cocktail (Promega), followed by incubation with 50 µl anti-Flag magnetic beads or anti-mouse IgG agarose beads (Sigma-Aldrich) for 12 h at 4°C. The beads were then washed six times with wash buffer (20 mM HEPES, 300 mM NaCl, 1 mM EDTA, 0.1% NP-40, and 0.1% glycerol, pH 7.4) and subjected to WB analysis. RIP analysis was performed using Magna RIP RNA-Binding Protein Immunoprecipitation Kit (Millipore, Billerica, MA, USA) according to the manufacturer's instructions. Briefly, cells were UV-crosslinked with 100,000 µJ/cm^2 twice, lysed in RIP lysis buffer supplemented with 0.2% protease inhibitor Cocktail and 0.1% RNase inhibitor, and subjected to immunoprecipitation. The immunoprecipitated complex were digested with proteinase K, followed by RNA extraction and quantitation of VAL using qRT-PCR.

Endogenous RNA pull-down assays and mass spectrometry analysis. For endogenous RNA pull-down assay, biotinylated short probes against VAL or its antisense (used as negative control) were designed and synthesized as described in the smFISH assay. According to the Chang lab's protocol [2], cell pellets were prepared and resuspended in lysis buffer (50 mM Tris-HCl pH7.0, 3 mM EDTA, 1% SDS, 1 mM DTT, 1 mM PMSF, protease inhibitor, 0.1 U/ul Superase-in, 2 mM Vanadyl ribonucleoside complex) on ice for 10 min, sonicated using Bioruptor (Diagenode, Seraing, Belgium) for 2 min at 50 W in ice bath and then diluted in two times volume of hybridization buffer (50 mM Tris 7.0, 750 mM NaCl, 1% SDS, 1 mM EDTA, 15% Formamide, 1 mM DTT, 1 mM PMSF, protease inhibitor, 0.1 U/ul Superase-in, 2mM Vanadyl ribonucleoside complex), followed by addition of 100 pmol biotinylated probes for hybridization by end-to-end rotation at 37°C for 4 h. Dynabeads Streptavidin C1 beads (Sigma-Aldrich) were incubated with the whole above mixture and captured by magnets. The beads biotinylated probes-RNAs-proteins adducts were washed five times with wash buffer, resuspended in DNase buffer, and eluted for bound proteins with Elution Buffer. At last, eluted proteins were

separated by SDS-PAGE and manifested by silver staining. Distinct bands of both VAL-interacting or VAL antisense-interacting proteins located at the same molecular weight in the gels were separately cut and subjected to mass spectrometry (MS) analysis (Applied Protein Technology, Shanghai, China). Notably, although the endogenous RNA pull-down assays for identifying VAL-interacting proteins were repeated three times, which similarly revealed distinct bands as manifested by silver staining, MS analysis of both VAL-interacting or VAL antisense-interacting proteins was done without biological or technical replicates. The proteins identified by MS analysis were further confirmed by endogenous RNA pull-down and Western Blots assays.

Exogenous RNA pull-down assays. For exogenous RNA pull-down assay, VAL RNAs were *in vitro* transcribed using T7 High Yield RNA Synthesis Kit (Ambion, Austin, TX, USA) according to the manufacturer's instructions. The transcribed RNAs were labeled with biotin using RNA 3' End Biotinylation Kit (Thermo, Waltham, MA, USA), and their binding proteins were enriched with an RNA-protein pull-down kit (Thermo, Waltham, MA, USA) as instructed. The protein-RNA-beads mixture was washed with 1× wash buffer for three times and eluted with Elution Buffer, and the retrieved RNA-interactive proteins were examined by WB analysis. Antisense RNAs of VAL were *in vitro* transcribed, biotinylated and used as a negative control.

Chromatin immunoprecipitation (ChIP) assay. ChIP analysis was performed with a commercially available ChIP Assay Kit (Upstate Biotechnology, Lake Placid, NY, USA) by following the manufacturer's instructions. In brief, cells (3×10^6) cultured in 15 cm culture dishes were harvested for cross-linking and sheared by sonication. The resultant chromatin fraction was immunoprecipitated using anti-STAT3 (Cell Signaling, #9139) or negative control anti-IgG (Sigma-Aldrich) antibodies. After reversing the cross-links with NaCl and removing proteins with proteinase K, enriched DNA fragments were purified and isolated via phenol/chloroform extraction and ethanol precipitation. Real-time quantitative PCR of purified DNA fragments was performed with the

indicated specific primers designed based on the putative STAT3 binding site in the promoter/enhancer region of *LINC01546* (sense: GAGCTTCCTCTTCCTGTC, antisense: CTGGCAGAACTCAGTGAT).

Enzyme-linked immunosorbent assay (ELISA). A human IL6 ELISA kit was purchased from R&D Systems (D6050; Minneapolis, MN, USA). Cells were cultured in 6-well dishes for 48 h to reach proximately 80% confluence. The culture medium was then collected to measure the secreted IL6 levels according to the manufacturer's instructions.

Animal models. Female BALB/c-nu mice (5-6 weeks of age, 18-20 g) were housed in specific pathogen-free facilities for animal studies. At least five nude mice per group were used to ensure the adequate power and each mouse with different weight was randomly allocated. For establishment of subcutaneous invasion model, indicated cells (5×10^6) were subcutaneously implanted into the left- or right-side inguinal folds of nude mice (n = 5 per group). Tumor formation was monitored over a 4-week period and then the mice were sacrificed for tumor excision. Both the xenografted tumors and superficial subcutaneous tissues connected together were excised. Sections of skin tumors were stained with H&E to visualize the tumor structure and boundary. For intracardiac injection, the indicated luciferase-expressing cells with various cell numbers (5×10^5, 5×10^4 and 5×10^3) were resuspended in 0.1 ml PBS and inoculated into the left cardiac ventricle of nude mice. To evaluate the therapeutic effect of antagonizing VAL, one week after 5×10^5 indicated cells in 0.1 ml PBS were intracardially injected as mentioned above, mice received 10 nmol chemosynthetic VAL siRNAs or scramble siRNAs as negative control two times a week via tail vein injection. Metastases were monitored by bioluminescent imaging every 3 days. For orthotopic implantation [3], the indicated luciferase-expressing cells (5×10^4) were mixed with matrigel (1:1) in a final volume of 50 µl cell suspension, and were injected quickly by advancing an insulin syringe equipped with a 29G × 1/2" needle to a depth of approximately 0.6 cm into the intercostal space

between the 5th and 6th ribs of the right lung of nude mice. For bioluminescent imaging assay, fifteen minutes prior to imaging, mice were injected intraperitoneally (i.p.) with 150 mg/kg luciferin. Following general anesthesia, images were taken and analyzed with Spectrum Living Image version 4.2 (Caliper Life Sciences, Waltham, MA, USA). At the experimental endpoint, nude mice were anesthetized and sacrificed, and various organs (brain, lung, liver, and bones) were resected, sectioned (5 μm in thickness) and histologically examined by H&E staining. All experimental procedures were approved by the Institutional Animal Care and Use Committee of Sun Yat-sen University.

References:

1. Raj, A., van den Bogaard, P., Rifkin, S.A., van Oudenaarden, A. & Tyagi, S. Imaging individual mRNA molecules using multiple singly labeled probes. *Nat Methods* **5**, 877-9 (2008).
2. Chu, C. et al. Systematic discovery of Xist RNA binding proteins. *Cell* **161**, 404-16 (2015).
3. Justilien, V. & Fields, A.P. Utility and applications of orthotopic models of human non-small cell lung cancer (NSCLC) for the evaluation of novel and emerging cancer therapeutics. *Curr Protoc Pharmacol* **62**, 14 27 1-14 27 17 (2013).
