# Supplementary material for: AKT-induced lncRNA VAL promotes EMT-independent metastasis through diminishing Trim16-dependent Vimentin degradation
Source: Nat Commun. 2020 Oct 12;11:5127. doi: 10.1038/s41467-020-18929-0 (PMC7550350; doi:10.1038/s41467-020-18929-0)
Supplement: Supplementary file 3 — Description of Additional Supplementary Data Files [file 41467_2020_18929_MOESM3_ESM.docx]

**Description of Additional Supplementary Data Files**

**Supplementary Data 1. Myr-AKT1-induced genes in LAD cells.** The significant altered genes (log2 Fold change > 2 or < -2) in myr-AKT1-overexpressing A549 or HCC827 cells, as compared to their corresponding vector-control cells are listed. Gene expression data were derived from strand-specific sequencing of polyA-enriched RNAs. Data were analyzed by the Wald Chi-Squared Test and adjusted P values were derived by Benjamini-Hochberg method. Original sequencing data have been deposited in GEO database with accession number GSE136904.

**Supplementary Data 2. VAL-interacting proteins identified by mass spectrometry analysis.** The unique peptide counts of both VAL- and VAL antisense-interacting proteins from mass spectrometry analysis of endogenous VAL RNA pull-down assays performed with biotinylated sense or antisense probes against VAL.
