# Supplementary material for: AKT-induced lncRNA VAL promotes EMT-independent metastasis through diminishing Trim16-dependent Vimentin degradation
Source: Nat Commun. 2020 Oct 12;11:5127. doi: 10.1038/s41467-020-18929-0 (PMC7550350; doi:10.1038/s41467-020-18929-0)
Supplement: Supplementary file 6 — Reporting Summary [file 41467_2020_18929_MOESM6_ESM.pdf]

nature research

Corresponding author(s): Junchao Cai

Last updated by author(s): Sep 7, 2020

Reporting Summary

Nature Research wishes to improve the reproducibility of the work that we publish. This form provides structure for consistency and transparency in reporting. For further information on Nature Research policies, see our Editorial Policies and the Editorial Policy Checklist.

Statistics

For all statistical analyses, confirm that the following items are present in the figure legend, table legend, main text, or Methods section.

n/a	Confirmed	
☐	☒	The exact sample size (*n*) for each experimental group/condition, given as a discrete number and unit of measurement
☐	☒	A statement on whether measurements were taken from distinct samples or whether the same sample was measured repeatedly
☐	☒	The statistical test(s) used AND whether they are one- or two-sided<br>*Only common tests should be described solely by name; describe more complex techniques in the Methods section.*
☒	☐	A description of all covariates tested
☒	☐	A description of any assumptions or corrections, such as tests of normality and adjustment for multiple comparisons
☐	☒	A full description of the statistical parameters including central tendency (e.g. means) or other basic estimates (e.g. regression coefficient) AND variation (e.g. standard deviation) or associated estimates of uncertainty (e.g. confidence intervals)
☒	☐	For null hypothesis testing, the test statistic (e.g. *F*, *t*, *r*) with confidence intervals, effect sizes, degrees of freedom and *P* value noted<br>*Give P values as exact values whenever suitable.*
☒	☐	For Bayesian analysis, information on the choice of priors and Markov chain Monte Carlo settings
☒	☐	For hierarchical and complex designs, identification of the appropriate level for tests and full reporting of outcomes
☒	☐	Estimates of effect sizes (e.g. Cohen's *d*, Pearson's *r*), indicating how they were calculated

Our web collection on statistics for biologists contains articles on many of the points above.

Software and code

Policy information about availability of computer code

Data collection	EPICS XL-MCL flow cytometer with EXPO32 ADC software version 1.2 (Beckman Coulter, Brea, CA, USA) for DNA content analysis; FISH images were obtained with a LSM810 confocal microscope using ZEN 2012 software version 8.1 (Carl Zeiss, Oberkochen, Germany); bioluminescent images were taken and analyzed with Spectrum Living Image version 4.2 (Caliper Life Sciences, Waltham, MA, USA).
Data analysis	Statistical analyses were performed using the PASW Statistics 18 version 18.0.0 (SPSS Inc., Chicago, IL, USA) software package and GraphPad Prism 8 version 8.3.0 (GraphPad Software, San Diego, CA, USA).

For manuscripts utilizing custom algorithms or software that are central to the research but not yet described in published literature, software must be made available to editors and reviewers. We strongly encourage code deposition in a community repository (e.g. GitHub). See the Nature Research guidelines for submitting code & software for further information.

Data

Policy information about availability of data

All manuscripts must include a data availability statement. This statement should provide the following information, where applicable:

- Accession codes, unique identifiers, or web links for publicly available datasets
- A list of figures that have associated raw data
- A description of any restrictions on data availability

The TCGA Lung Adenocarcinoma (TCGA-LUAD) sequencing data used in this study are available in a public repository from the GDC Data Portal (https://portal.gdc.cancer.gov/). The RNA-sequencing data that support the findings of this study has been deposited in GEO with the accession code GSE136904. The source data underlying Figs. 1b, d-l, n, 2a-f, 3b-d, i, 4b, c, e, f, 5a-i, 6a-j, 7a and e, and Supplementary Figs. 1b-d, f-k, 2e-g, 3a, c, d, f-i, l, 4a-d, f, g, 5a, b, d-j, 6a-f, h-l and n are provided as a Source Data file. All other data supporting the findings of this study are available from the corresponding author upon reasonable request.

Field-specific reporting

Please select the one below that is the best fit for your research. If you are not sure, read the appropriate sections before making your selection.

☒ Life sciences ☐ Behavioural & social sciences ☐ Ecological, evolutionary & environmental sciences

For a reference copy of the document with all sections, see nature.com/documents/nr-reporting-summary-flat.pdf

Life sciences study design

All studies must disclose on these points even when the disclosure is negative.

Sample size	Sample size was determined by power analysis to achieve a minimum effect size of 0.5 with a P value of less than 0.05 and all sample sizes were appropriate for assumption of normal distribution.
Data exclusions	Clinical specimens histopathologically observed without cancerous lesions and animals bearing tumors in sizes larger than 2cm were excluded from the analysis. The criteria were pre-established.
Replication	All experiments were repeated at least three times with reproducibility. Specifically, the replication number for each experiment is indicated in the legend of the corresponding figure.
Randomization	Cell line experiments: For each experiment, the total amount of cells from one cell line required for all tested conditions were pooled and seeded randomly into different plates, pre-labeled with the treatment to be applied.<br>Mice experiments: For the in vivo experiments, mice were randomly put into cages and randomly assigned to experimental groups.
Blinding	For the imaging experiments, where manual counting was required, samples were labeled with numbers in order to avoid preconceptions of the analyzing investigator. For western blot, codification was maintained during sample preparation and decoding was done before loading the samples on the SDS-PAGE gel, in order to ensure an adequate presentation of the results.

Reporting for specific materials, systems and methods

We require information from authors about some types of materials, experimental systems and methods used in many studies. Here, indicate whether each material, system or method listed is relevant to your study. If you are not sure if a list item applies to your research, read the appropriate section before selecting a response.

Materials & experimental systems

n/a	Involved in the study	
☐	☒	Antibodies
☐	☒	Eukaryotic cell lines
☒	☐	Palaeontology and archaeology
☐	☒	Animals and other organisms
☐	☒	Human research participants
☒	☐	Clinical data
☒	☐	Dual use research of concern

Methods

n/a	Involved in the study	
☒	☐	ChIP-seq
☒	☐	Flow cytometry
☒	☐	MRI-based neuroimaging

Antibodies

Antibodies used	Primary antibodies used for WB analysis were: anti-Vimentin (Proteintech, 60330-1-Ig, 1:2000), anti-AKT1 (Proteintech, 10176-2-AP, 1:800), anti-phosphor AKT1 (S473) (Proteintech, 66444-1-Ig, 1:800), anti-phosphor STAT3 (S727) (Cell Signaling Technology, #34911, 1:400), anti-STAT3 (Cell Signaling, #9139, 1:800), anti-E-Cadherin (Cell Signaling, #3195, 1:400), anti-N-Cadherin (Cell Signaling, #13116, 1:400), anti-γ-Catenin (Abcam, ab184919, 1:800), anti-Trim16 (Santa Cruz, sc-398851, 1:800), anti-FLAG (Sigma-Aldrich, F1804, 1:2000), anti-HA (Sigma-Aldrich, H3663, 1:2000), anti-MYC (Proteintech, 16286-1-AP, 1:2000) and Normal Mouse IgG for immunoprecipitation (Sigma-Aldrich, 12-371). Blotted membranes were stripped and re-blotted with anti-GAPDH (Proteintech, 10494-1-AP, 1:2000) used as loading controls.
Validation	Antibodies were chosen based on previous literature. Validation and quality control is available from the manufacturers using the catalog number of each antibody. When necessary, additional validations were performed in our laboratory using siRNA- or shRNA- treated cells for the depletion of the targeted protein.<br><br>anti-Vimentin (Proteintech, 60330-1-Ig): Chen Y, et al. Mol Cancer. 2019 Jan 4;18(1):1.<br><br>anti-AKT1 (Proteintech, 10176-2-AP): Yang T, et al. Oncogene. 2018 Nov;37(45):5997-6009.<br><br>anti-phosphor AKT1 (S473) (Proteintech, 66444-1-Ig): Zhang Q, et al. Oncotarget. 2015 Oct 13;6(31):32177-92.

anti-phosphor STAT3 (S727) (Cell Signaling Technology, #34911): Li C, et al. J Clin Med. 2019 Sep 5;8(9):1391.

anti-STAT3 (Cell Signaling, #9139): Wu Q, et al. Cell Death Dis. 2020 Feb; 11(2): 131.

anti-E-Cadherin (Cell Signaling, #3195): Tian Q, et al. Oncogene. 2020 May;39(20):3980-3996.

anti-N-Cadherin (Cell Signaling, #13116): Raoof S, et al. Oncogene. 2019 Sep;38(37):6399-6413.

anti-γ-Catenin (Abcam, ab184919): Civciristov S, et al. J Biol Chem. 2019 Nov 1;294(44):16198-16213.

anti-Trim16 (Santa Cruz, sc-398851): mouse monoclonal antibody specific for an epitope mapping between amino acids 181-206 within an internal region of TRIM16 of human origin, which is recommended for detection of TRIM16 of mouse, rat and human by Western Blotting and immunoprecipitation.

anti-FLAG (Sigma-Aldrich, F1804): He W, et al. Mol Cell. 2020 Apr 2;78(1):168-183.

anti-HA (Sigma-Aldrich, H3663): Lyon K, et al. Mol Cell. 2019 Jul 11;75(1):172-183.

anti-MYC (Proteintech, 16286-1-AP): Ji S, et al. Dev Cell. 2019 Feb 25;48(4):460-474.

Normal Mouse IgG for immunoprecipitation (Sigma-Aldrich, 12-371): Kim J, et al. Nat Struct Mol Biol. 2019 Mar;26(3):213-219.

anti-GAPDH (Proteintech, 10494-1-AP): Li K, et al. Cell Res. 2020 Feb;30(2):163-178.

Eukaryotic cell lines

Policy information about cell lines

Cell line source(s)	LAD cell lines, including A549, HCC827, H1650, H596, H1975, H1299, H292, H2009 and H2030 and non-cancerous HEK293FT, HFL1 and MRC5 cells were purchased from the American Type Culture Collection (ATCC; Manassas, VA, USA) and maintained in Dulbecco's modified Eagle's medium (DMEM for LAD cell lines and HEK293FT cell; GIBCO, Carlsbad, CA, USA), Improved Minimum Essential Medium (IMEM for MRC5 cell; GIBCO, Carlsbad, CA, USA) or Ham's F-12K Medium (for HFL1 cell; GIBCO, Carlsbad, CA, USA) supplemented with 10% fetal bovine serum (FBS; Corning, Tewksbury, MA, USA) and 1% penicillin/streptomycin (penicillin 100 U/ml and streptomycin 10 μg/ml) (GIBCO). LAD3 cells were isolated from the tumor biopsy collected from a stage III LAD patient. The Beas2B immortalized human bronchial epithelial cell line obtained from ATCC was cultured in Bronchial Epithelial Cell Growth Medium (BEGM) as instructed by the supplier (Lonza, Walkersville, MD, USA).
Authentication	All cell lines were authenticated by short tandem repeat (STR) fingerprinting at Medicine Laboratory of Forensic Medicine Department of Sun Yat-Sen University (SYSU) (Guangzhou, China).
Mycoplasma contamination	All cell lines were tested to be free of mycoplasma contamination.
Commonly misidentified lines (See ICLAC register)	No commonly misidentified cell lines were used in the study.

Animals and other organisms

Policy information about studies involving animals; ARRIVE guidelines recommended for reporting animal research

Laboratory animals	BALB/c-nu female mice (5-6 weeks of age, 18-20 g)
Wild animals	Study did not involve wild animals.
Field-collected samples	Study did not involve field-collected samples.
Ethics oversight	All of the animal procedures were approved by the Sun Yat-sen University Animal Care Committee.

Note that full information on the approval of the study protocol must also be provided in the manuscript.

Human research participants

Policy information about studies involving human research participants

Population characteristics	All clinical tissue specimens used in this study were obtained from and histopathologically diagnosed as lung adenocarcinoma at Sun Yat-Sen Memorial Hospital. There are no age or gender restrictions.
Recruitment	34 LAD patients' tumor tissues and adjacent non-tumorous tissues were collected consecutively at Sun Yat-Sen Memorial Hospital without artificial selection bias.
Ethics oversight	Prior patients' consent and approval from the Institutional Research Ethics Committee of Sun Yat-sen University were obtained for the use of these clinical materials for research purposes. The study is compliant with all relevant ethical

regulations involving human participants.

Note that full information on the approval of the study protocol must also be provided in the manuscript.
