# Supplementary material for: AKT-induced lncRNA VAL promotes EMT-independent metastasis through diminishing Trim16-dependent Vimentin degradation
Source: Nat Commun. 2020 Oct 12;11:5127. doi: 10.1038/s41467-020-18929-0 (PMC7550350; doi:10.1038/s41467-020-18929-0)
Supplement: Supplementary file 2 — Peer Review File [file 41467_2020_18929_MOESM2_ESM.docx]

**Reviewers' comments:**

**Reviewer #1 (Remarks to the Author):**

In the present study, the authors found that LINC01546 is induced by AKT/STAT3 signaling in lung adenocarcinoma cells and promotes metastasis via proteins stabilization of vimentin. LINC01546 protects vimentin from Trim16-mediated downregulation. The authors stated that the whole process does not accompany epithelial-mesenchymal transition. Animal experiments were well performed.

LINC01546 has been only poorly characterized thus far. The finding that LINC01546 mediates AKT-induced cell invasiveness is novel and potentially interesting. It can be a novel molecular target for anti-cancer therapy. However, I have a few concerns as follows:

**1)** My major criticism is that the authors exclusively used overexpression systems to supports conclusions. I feel that the situation in which myr-AKT1 or LINC01546 is overexpressed is very artificial. The authors should examine the roles of LINC01546 and Trim16 in lung cancer cells with activating PIK3CA or AKT mutation (H1975 etc.).

**Response:** We greatly appreciate the reviewer’s encouraging comment. The authors well understand the reviewer’s concern and have taken this suggestion seriously. In this revision, a LAD cell line H1975 carrying the activating *PIK3CA* (G118D) mutation and expressing high levels of LINC01546, which is named as VAL short for **v**imentin-**a**ssociated **l**ong noncoding RNA in this revised manuscript, was employed to evaluate the roles of VAL (LINC01546) and Trim16 with a series of *in vitro* and *in vivo* experiments. As shown in **new Fig. 2d-f**, silencing VAL markedly abrogated the abilities of H1975 cells to bind to and invade through the extracellular matrix and promoted suspension-induced anoikis. Moreover, silencing VAL drastically abrogated the abilities of H1975 cells to form systemic metastases, as evidenced by the compromised systemic bioluminescent signals, almost intact bone tissue without cancerous lesion, and prolonged metastasis-free and overall survival of nude mice intracardially injected with VAL-silenced H1975 cells as compared to control vector-transduced H1975 cells (**new Fig. 2h-j**). Also in our attempt to address Reviewer 3’s request by employing an orthotopic implantation model, we found that silencing VAL in H1975 cells, or in myr-AKT1-overexpressing LAD cells as well, significantly suppressed intra-pulmonary metastases (**new Supplementary Fig. 3k-m**). At the molecular level, silencing VAL in H1975 cells significantly accelerated K48-linked polyubiquitination and degradation of Vimentin proteins and reduced protein levels but not the mRNA levels of Vimentin (**new Fig. 5e, f and i**). Furthermore, silencing VAL failed to cause Vimentin protein polyubiquitination or decrease Vimentin protein levels when Trim16 was pre-silenced (**new Supplementary Fig. 5i, j**). Similar to the consequence of overexpression of Vimentin or restoration of shRNA-resistant version of VAL, knockdown of Trim16 greatly reserved the inhibitory effect of silencing VAL on adhesion, invasion or anoikis resistance of H1975 cells (**new Fig. 7e, Supplementary Fig. 6k, l**). These data further demonstrate in an endogenous PI3K/AKT activation scenario the pro-metastatic role of VAL dependent on abrogation of Trim16-induced Vimentin degradation. Corresponding results have been incorporated into the revised manuscript.

**2)** The authors stated that constitutively active AKT1 enhances invasive properties of A549 and HCC827 “without inducing EMT” (line 124-125). But they only examined cell morphology. This is not enough. They should examine other EMT-associated phenotypes including cadherin switching.

**Response:** The authors thank the reviewer for this suggestion. In consistence with the data showing that constitutively active AKT1 failed to induce morphological changes of A549 and HCC827 cells, expression of epithelial and mesenchymal cell markers such as E-cadherin, γ-catenin and N-cadherin, as well as EMT-related transcriptional factors such as Snail, Slug, Twist, ZEB1 and ZEB2, were hardly affected by constitutively active AKT1 in these LAD cells (**new Supplementary Fig. 1a-c**). Similarly, overexpressing or silencing VAL in LAD cells neither induced morphological changes nor caused alterations in the expression of celluar EMT markers or transcriptional factors (**new Supplementary Fig. 3a-f**). In conjunction with the data showing that constitutively active AKT1-induced VAL promotes metastasis through binding and stabilizing Vimentin proteins in LAD cells, these results suggest that AKT1 overactivation and upregulated levels of VAL are able to potentiate invasive properties of LAD cells through upregulating Vimentin levels without inducing the EMT phenotype. Corresponding results have been incorporated into the revised manuscript on Pages 6 and 9.

**3)** The authors should discuss physiological roles of this regulatory system for vimentin stability. Such discussion would attract a wider readership. Are LINC01546 and Trim16 expressed in mesenchymal cells? I wonder if Trim16-mediated vimentin downregulation is a system for epithelial cells.

**Response:** The authors appreciate that the reviewer has raised an interesting point. As VAL has no homolog molecule in mouse, in this revision, we measured expression levels of VAL and Trim16 in two human foetal lung fibroblast cell lines, namely, HFL1 and MRC5. As shown in **Supplementary Table 2** and **Supplementary Fig. 1a only for Reviewer**, the absolute copy number of VAL in HFL1 or MRC5 is nearly zero per cell, indicating that VAL expression is almost undetectable in these mesenchymal cells. By contrast, protein levels of Vimentin in mesenchymal cells (HFL1 and MRC5) are higher than those in non-cancerous epithelial cells Beas2B and cancerous epithelial cells A549 and HCC827; protein levels of Trim16 in mesenchymal cells (HFL1 and MRC5) are similar to those in both non-cancerous and cancerous cells of epithelial origin (Beas2B, A549, and HCC827), indicating that both Trim16 and Vimentin are normally expressed in mesenchymal cells (**Supplementary Fig. 1b only for Reviewer**). Moreover, overexpressing VAL or Trim16 in MRC5 cells failed to alter protein levels of Vimentin, whereas overexpressing VAL increased and overexpressing Trim16 decreased Vimentin expression in Beas2B cells, similar to the effects of VAL in LAD cells as shown in the manuscript (**Supplementary Fig. 1c only for Reviewer**). Also notably, we found that the expression levels of VAL or Trim16 were barely altered in TGF-β-overexpressing A549 cells undergoing mesenchymal morphology changes as compared to vector-control A549 cells whereas both mRNA and protein levels of Vimentin were significantly upregulated in TGF-β-induced mesenchymal cells of A549, accompanied with alterations of other EMT cell markers accordingly (**Supplementary Fig. 1, d-f only for Reviewer**), indicating that in a scenario of EMT process, Vimentin expression is not regulated by VAL, whereas is more likely to be regulated at the transcriptional level, such as by EMT-related transcription factors. In light of these results together, we propose that the VAL-Vimentin-Trim16 interaction model is a unique system for epithelial cells. Appropriate description has been added to the **Discussion** section of the revised manuscript on Page 19.

**Reviewer #2 (Remarks to the Author):**

AKT plays a role in the induction of various cancer, but the mechanism by which it operates and the frequent lack of response to inhibitors are not understood as yet. In this current study, the authors identified a long non-coding RNA (lncRNA) LINC01546, which is directly induced by AKT/STAT3 signaling. siRNAs against LINC01546 shows great therapeutic potential in metastatic cells driven by AKT overactivation. This may be induced by a direct binding to Vimentin that stabilizes this protein. Overall, this is a well-written and interesting manuscript that demonstrate for the first time lncRNA-mediated mechanism for AKT-driven metastasis. In addition, these findings may lead to new strategies to target metastatic LAD patients. However, I do have several comments and suggestions that should be addressed in order to allow publication. These are as follows:

**Major comments:**

**1.** In ENCODE RNA-seq data, the levels of LINC01546 appear to be very low in A459 cells. The authors observe an induction following AKT activation, but what are the levels (in FPKM units and in estimated copy number per cell, ideally matching the number of dots the authors see per cell in their single-molecule FISH images) in the induced cells? The proposed model is that this lncRNA binds Vimentin, which is an abundant protein. For the model in which the RNA stabilizes Vimentin to make sense, lncRNA copy number should be at the same order of magnitude as Vimentin. Related to this – what is the copy number of LINC01546 in the over-expressing cells (Fig. 2a-c)? How does it compare to physiological levels of LINC01546 in AKT-activated cells?

**Response:** The authors thank the reviewer for the encouraging comment and appreciate that the reviewer has raised an issue of great importance. In ENCODE RNA-seq data, the levels of LINC01546 in FPKM units are 0.3 in A549 cells. However, in our study, using the Poly(A) RNA Selection Module that enables highly specific enrichment of polyadenylated RNAs from total RNA samples, the strand specific RNA sequencing analysis showed that FPKM units of LINC01546 (ENSG00000228459, which is named as VAL for short for **v**imentin-**a**ssociated **l**ong noncoding RNA in this revised manuscript) are 4.37 in A549-Vector cells, 128.25 in A549-myr-Akt1 cells, 3.23 in HCC827-Vector cells, and 75.50 in HCC827-myr-Akt1 cells (GSE136904 as provided by our study). The absolute quantification assay showed that the estimated copy number per cell of VAL is 41, 937, 34 and 768 copies, respectively, for A549-Vector, A549-myr-Akt1 cells, HCC827-Vector, and HCC827-myr-Akt1 cells (**Supplementary Table 2**), which appear to match the number of dots per cell shown in the single-molecule FISH images.

On the other hand, to calculate the absolute level of Vimentin proteins, a serial dilution of purified Vimentin proteins was used for Western Blotting to generate a standard reference. The mass of purified Vimentin proteins was calculated by the formula: (equivalent molecules/cell) × (2×10^4^ cells) / (6.02×10²³, NA) × (molecular weight of Vimentin, 57KDa), and 0.38pg, 0.76pg, 1.51pg, 3.02pg and 4.54pg of purified Vimentin proteins were loaded, which are approximately equivalent to 2×10^2^, 4×10^2^, 8×10^2^, 16×10^2^ and 24×10^2^ molecules per cell. Subsequently, 6μg of protein lysates of HCC827, A549, and H1975 cells (2×10^4^ cells × 300pg protein/cell) were loaded. Following Western Blotting analysis, relative protein levels of immunoblots were quantified by scanning densitometry. According to the relative protein intensity, it is estimated that there are approximately 280, 750, and 1580 molecules of Vimentin proteins per cell, respectively, in HCC827, A549, and H1975 cells, in which the estimated copy numbers per cell of VAL are 34, 41, and 167, indicating that although the copy number of VAL is close to the order of magnitude as that of Vimentin (less than or slightly more than 10 folds), there are a bit more molecules of Vimentin proteins than VAL (**Supplementary Fig. 1g only for Reviewer and Supplementary Table 2**). Notably, we found that half-lives of VAL molecules are much longer than those of Vimentin proteins in the same LAD cells and constitutively activated AKT slightly promoted RNA stabilization of VAL (**Supplementary Fig. 1h only for Reviewer**), indicating that long half-life of VAL might also contribute to the stabilization and upregulation of Vimentin proteins in LAD cells.

The absolute amounts of VAL were 41 vs 386, 21 vs 186, 167 vs 15 and 23 copies per cell in A549-Vector vs A549-VAL, H2009-Vector vs H2009-VAL, and H1975-shVector vs H1975-VAL-sh1 and H1975-VAL-sh2 cells, respectively. In parallel, the absolute quantities of VAL were approximately 54-93 copies, 119-188 copies, 206-243 copies, and were 324-576 copies per cell, respectively, in LAD tissues derived from patients of stage I, II, III, IV (**Supplementary Table 2**). Notably, the copy number of VAL in a stage III LAD tissue carrying the activating *PIK3CA*(E545K) mutation was 908, which is similar to the absolute level of VAL in LAD cells overexpressing myr-AKT1 (**Supplementary Table 2**). By contrast, there were only 2-7 copies per cell in normal lung tissue derived from LAD patients of stage I, similar to the absolute level of VAL in human normal lung epithelial cells Beas2B (**Supplementary Table 2**). Therefore, the absolute VAL levels in VAL-overexpressing cells are within the range of those in human NSCLC tissue samples, especially with *PIK3CA* activation mutation and were much higher than the physiological VAL levels in normal lung cells and tissues. Corresponding results have been incorporated into the revised manuscript on Pages 8 and 9.

**2.** If the lncRNA works in trans by stabilizing Vimentin, can the authors rescue the KD phenotype by expressing an shRNA-resistant version of the lncRNA sequence?

**Response:** In this revision, we constructed an shRNA-resistant mutant of VAL according to the sequence of the shRNA#1 and evaluated its rescue effect on cell invasion, adhesion, and anoikis resistance in H1975 cells transduced with the specific shRNA#1. As shown in **new Fig. 7e and Supplementary Fig. 6k-n**, similar to the consequence of Vimentin overexpression Trim16 depletion, restoring the shRNA-resistant mutant of VAL could fully reverse the inhibitory effects of silencing VAL on cell invasion, adhesion and anoikis resistance, whereas overexpression of wild-type VAL, which mildly restored VAL levels in VAL-silenced H1975 cells, slightly potentiated the abilities of VAL-silenced H1975 cells to adhere to or invade through the Matrigel, or resist anoikis. Taken together, our data suggest that VAL indeed works in trans by stabilizing Vimentin to promote LAD metastasis. Corresponding results have been incorporated into the revised manuscript on Page 15.

**3.** The authors used myr-AKT as a constitutively active isoform. However, this construct is mis-localized, and therefore its effect might be distinct from that of the endogenous AKT isoforms. It would be interesting to examine the effect of acute short-term stimulation (e.g. by insulin) on the induction of LINC01546, and compare it to the one induced by constitutive stimulation.

**Response:** The reviewer’s concern is well understood. In this revision, we employed acute short-term stimulation together with inhibitor treatments to examine the effect of AKT1/STAT3 activity on the induction of VAL (LINC01546). As shown in **new Supplementary Fig. 1f, g**, transient stimulation of A549 and HCC827 cells with recombinant insulin or FGF2, which markedly activated AKT1 but slightly activated STAT3 in these LAD cells, mildly induced VAL expression, the degrees of increase in which are much smaller than the one induced by constitutively activated AKT. Moreover, treatment with AKT inhibitor (MK-2206), or STAT3 inhibitor (S3I-201) could completely abrogate the promoting effect of insulin or FGF2 stimulation on VAL expression (**new Supplementary Fig. 1h**). Similarly, treatment of H1975, a LAD cell line carrying the activating mutant *PIK3CA*(G118D) and expressing high levels of VAL, with AKT inhibitor, or STAT3 inhibitor also markedly decreased VAL expression (**new** **Fig. 1b**). Furthermore, also per Reviewer 3’s suggestion, using a Tet-on inducible system in H1975 cells, addition of doxycycline (Dox) caused transient silencing of AKT1 and greatly decreased VAL expression, and both IL6 stimulation and overexpression of constitutively active STAT3 reversely upregulated VAL expression in H1975 cells silenced with AKT1 by Dox addition (**new Supplementary Fig. 1j, k**). These data support the potent promoting effect of AKT overactivation on the induction of VAL expression and the importance of STAT3 activity in AKT overactivation-induced high levels of VAL. Corresponding data have been incorporated into the revised manuscript on Pages 6 and 7.

**4.** The activation of STAT3 downstream of AKT can occur by several distinct pathways. Some information on the pathway involve in the studied cells, and comparison with short term stimulation may clarify the signaling involved in this system, and the way it affect vimentin’s ubiquitination.

**Response:** As mentioned above, short term stimulation of LAD cells with recombinant insulin or FGF2 apparently activated AKT and mildly increased VAL expression. However, the increasing folds of VAL expression induced by insulin or FGF2 stimulation are much smaller than the one induced by constitutively active AKT, which should be ascribed to the slight activation of STAT3 stimulated by insulin or FGF2 (**new Fig. 1b, and Supplementary Fig. 1f, g**). In consistence, insulin or FGF2 treatment faintly decreased polyubiquitination of Vimentin proteins and thus marginally increased Vimentin protein levels (**new Supplementary Fig. 5g, h**), which was strikingly diminished by overexpression of constitutively active AKT in A549 cells, or was quite low level in H1975 cells carrying the activating *PIK3CA*(G118D) mutation (**new Fig. 1b and Supplementary Fig. 5i**), indicating the importance of VAL induced by fully activated AKT/STAT3 signaling in the process of Vimentin upregulation. Moreover, treatment with AKT or STAT3 inhibitor completely abrogated the promoting effect of short term stimulation or constitutive activation of AKT on the induction of VAL; notably, mTOR inhibitor could greatly reverse the promoting effect of insulin or FGF stimulation on VAL expression but mildly decrease VAL expression in LAD cells overexpressing constitutively active AKT (**new Fig. 1d and Supplementary Fig. 1h**). Interestingly, we found that IL6 was significantly upregulated in LAD cells overexpressing constitutively active AKT at both the transcription and secretion levels, and that NF-κB inhibitor failed to reverse the promoting effect of constitutively active AKT on IL6 expression, whereas short term stimulation with insulin or FGF2 barely affected IL6 expression in LAD cells (**new Fig. 1g, h and Supplementary Fig. 1g**). Furthermore, stimulation with recombinant IL6 protein, which is a potent inducer of STAT3 activity, in LAD cells also significantly induced VAL expression, and STAT3 inhibitor greatly reversed IL6-induced expression of VAL while AKT inhibitor slightly compromised the effect of IL6 stimulation on VAL expression (**new Supplementary Fig. 1i**). In parallel, both IL6 stimulation and overexpression of constitutively active STAT3 reversely upregulated VAL expression in H1975 cells silenced with AKT1 by Dox addition using the Tet-on inducible system (**new Supplementary Fig. 1j, k**). Taken together, our data support the notion that constitutively activated AKT promotes STAT3 activity through multiple pathways, at least including NF-κB-independent IL6 transcription and mTOR pathways. Corresponding data have been incorporated into the revised manuscript on Pages 6, 7 and 13.

**Minor comments:**

**1.** Is the expression of the adjacent genes, PRKX, MXRA5, and ARSF up-regulated in cancer and affected by LINC01546 shRNAs?

**Response:** In the TCGA LAD datasets, PRKX and ARSF mRNA levels are marginally down-regulated in caner tissue, and MXRA5 mRNA levels are mildly up-regulated in cancer tissue, as compared to normal tissue (**new Supplementary Fig. 5a**). In addition, silencing VAL did not affect expression of PRKX, MXRA5 or ARSF (**new Supplementary Fig. 5b**), suggesting that VAL might not function as a cis-acting gene that often regulates transcription of neighbor genes.

**2.** For the RNA pulldown – please indicate how many probes were used and what were their sequences.

**Response:** For the endogenous RNA pull-down assay, 18 probes with different sequences covering almost the full-length VAL were used, and each of their sequences is provided in **Supplementary Table 3** in the revised manuscript.

**3.** Stabilization of Vimentin in Figure 5a – can this be quantified? How many times was this experiment repeated? Same for Figure 5f-g.

**Response:** In this revision, relative protein levels were quantified by scanning densitometry, and the relative gray value of proteins was calculated as Band Intensity of Protein of Interest / Band Intensity of Loading Control. As shown in the **new Figures 5 and 6**, the quantification values of immunoblots have been provided. These experiments were always repeated two times and the representative images derived from one of three independent experiments are presented. The corresponding information has been provided in the revised manuscript.

**4.** Line 253 – “polyubiquitination of Vimentin proteins was almost diminished” – should probably say “was markedly diminished”.

**Response:** This sentence has been corrected in the revised manuscript.

**5.** In figure 6I, it is suggested to replace the upper two blots, as the upper is torn, and in the second, the bands in the fourth lane are not straight.

**Response:** The authors thank the reviewer for pointing out the issue. New representative images of immunoblots in **new** **Fig. 6i** showing that knockdown of VAL failed to cause polyubiquitination of Vimentin proteins when Trim16 was pre-silenced in myr-AKT1-overexpressing LAD cells have been provided in the revised manuscript.

**Reviewer #3 (Remarks to the Author):**

In this manuscript, Tian and colleagues provide solid evidence that the lncRNA, LINC01546, which is induced by AKT/STAT3 signaling, promotes invasion and metastasis of lung cancer cells. This process does not seem to depend on EMT but is dependent on inhibition of vimentin degradation mediated through the E3 ubiquitin ligase Trim16 (also called estrogen-responsive B-box protein; EBBP). This is a ‘brand new’ intergenic lncRNA and the current study represents the first to investigate in detail on this lincRNA and links the X chromosome-localized LINC01546 to cancer metastasis. They show very interesting mechanistic data on direct binding of LINC01546 to vimentin thus blocking Trim16-mediated polyubiquitination and degradation of vimentin. Combined, this study demonstrates a lncRNA-mediated mechanism for AKT-driven (but perhaps EMT-independent) metastatic properties in lung cancer. Overall, most experiments were nicely executed, data are solid and support the conclusions drawn, the manuscript is succinctly and reasonably well-written. One major deficiency is the lack of some key studies in patient lung cancer samples.

**Major points:**

**1.** Supplementary Figure 1a-c: the claim on a lack of EMT in the two cell lines overexpressing myr-AKT1 should be backed up by molecular marker analysis such as E-/N-cadherin, vimentin, ZEB1/2 etc.

**Response:** All the authors greatly appreciate the reviewer’s encouraging comment and this suggestion. In consistence with the data showing that constitutively active AKT1 failed to induce morphological changes of A549 and HCC827 cells, expression of epithelial and mesenchymal cell markers such as E-cadherin, γ-catenin and N-cadherin, as well as EMT-related transcriptional factors such as Snail, Slug, Twist, ZEB1 and ZEB2, were hardly affected by constitutively active AKT1 in these LAD cells (**new Supplementary Fig. 1a-c**). Similarly, overexpressing or silencing LINC01546, which is named as VAL for short for **v**imentin-**a**ssociated **l**ong noncoding RNA in this revised manuscript, in LAD cells neither induced morphological changes nor caused alterations in the expression of EMT-related cell markers or transcriptional factors (**new Supplementary Fig. 3a-f**). In conjunction with the data showing that constitutively active AKT1-induced VAL promotes metastasis through binding and stabilizing Vimentin proteins in LAD cells, these results suggest that both AKT1 overactivation and upregulated levels of VAL are able to greatly enhance the invasive properties of LAD cells through upregulating Vimentin levels without inducing EMT phenotype. Corresponding results have been incorporated into the revised manuscript on Pages 6 and 9.

**2.** It is recommended that the authors present AKT-induced genes in a supplementary table.

**Response:** In this revision, the genes significantly induced (fold change ≥ 4, *P* < 0.05 ) by constitutively active AKT are provided in **Supplementary Table 1** in the revised manuscript. In addition, the sequencing data of vector-control and AKT1-overexpressing A549 or HCC827 cells have been deposited in GEO database with accession number GSE136904. Corresponding information has been incorporated into the revised manuscript on Page 6.

**3.** The critical conclusion that in LAD cells, AKT1 induces LINC01546 via STAT3 needs to be validated in some other cell models in Supplementary Figure 1k and using an inducible system (because stably selected cells may undergo many molecular changes).

**Response:** We thank the reviewer for raising this important point, and the suggestion is seriously taken. In this revision, also per Reviewer 2’s suggestion, we employed acute short-term stimulation together with inhibitor treatments and a Tet-on inducible system to examine the effect of AKT1/STAT3 activity on the induction of VAL (LINC01546). As shown in **Supplementary Fig. 1f-h**, transient stimulation of A549 and HCC827 cells with recombinant insulin or FGF2, which markedly activated AKT1 but slightly activated STAT3 in these LAD cells, mildly induced VAL expression, whereas AKT inhibitor (MK-2206), or STAT3 inhibitor (S3I-201) completely abrogated the promoting effect of insulin or FGF2 stimulation on VAL expression. Similarly, treatment of H1975, a LAD cell line carrying the activating *PIK3CA*(G118D) mutation and expressing high levels of VAL, with AKT or STAT3 inhibitor also greatly decreased VAL expression (**new Fig. 1b**). Interestingly, we found that IL6 was significantly upregulated in LAD cells overexpressing constitutively active AKT at both the transcription and secretion levels and NF-κB inhibitor failed to reverse the promoting effect of constitutively active AKT on IL6 expression, whereas short term stimulation with insulin or FGF2 barely affected IL6 expression in LAD cells (**new Fig. 1g, h and Supplementary Fig. 1g**). In consistence, stimulation with recombinant IL6 protein, which is a potent inducer of STAT3 activity, in A549 and HCC827 cells also significantly induced VAL expression, and STAT3 inhibitor greatly reversed IL6-induced expression of VAL while AKT inhibitor slightly compromised the effect of IL6 stimulation on VAL expression (**new Supplementary Fig. 1i**). Furthermore, using a Tet-on inducible system in Beas2B cells, addition of doxycycline (Dox) induced myr-AKT1 expression and markedly upregulated VAL expression, and further treatment with AKT or STAT3 inhibitors significantly reversed Dox-induced VAL expression (**new Fig. 1e, f**). In parallel, using a Tet-on inducible system in H1975 cells, addition of doxycycline (Dox) caused transient silencing of AKT1 and greatly decreased VAL expression, and both IL6 stimulation and overexpression of constitutively active STAT3 reversely upregulated VAL expression in H1975 cells silenced with AKT1 by Dox addition (**new Supplementary Fig. 1j, k**). Therefore, these data strongly suggest the potent promoting effect of AKT overactivation on the induction of VAL expression and the importance of STAT3 activity in AKT overactivation-induced high levels of VAL. Corresponding data have been incorporated into the revised manuscript on Pages 6 and 7.

**4.** Although human lung cancers clearly overexpress LINC01546 compared to normal tissues (Figure 1g-j), it’s not clear whether the levels the author artificially overexpressed in the lung cancer cell lines (Supplementary Figure 2a) were physiologically relevant.

**Response:** The authors greatly appreciate that the reviewer has raised an issue of great importance. The absolute quantification assay showed that the estimated copy number per cell of VAL was 41 vs 386, 21 vs 186, 167 vs 15 and 23 copies per cell in A549-Vector vs A549-VAL, H2009-Vector vs H2009-VAL, and H1975-shVector vs H1975-VAL-sh1 and H1975-VAL-sh2 cells, respectively (**Supplementary Table 2**). In parallel, the absolute quantities of VAL were approximately 54-93 copies, 119-188 copies, 206-243 copies, and were 324-576 copies per cell, respectively, in LAD tissues derived from patients of stage I, II, III, IV (**Supplementary Table 2**). Notably, the copy number of VAL in a stage III LAD tissue carrying an activating *PIK3CA* (E545K) mutation was 908, similar to the absolute VAL level in LAD cells overexpressing myr-AKT1 (**Supplementary Table 2**). By contrast, there were only 2-7 copies per cell in normal lung tissue derived from LAD patients of stage I, similar to the absolute level of VAL in human normal lung epithelial cells Beas2B (**Supplementary Table 2**). Therefore, the absolute VAL levels in VAL-overexpressing cells are within the range of those in human NSCLC tissue samples, especially with *PIK3CA* activation mutation and were far higher than physiological VAL levels in normal lung cells and tissues. Corresponding results have been incorporated into the revised manuscript on Pages 8 and 9.

**5.** Images shown in Figure 2d are not clear (authors should use arrows to demarcate the invasions) and data should be quantified.

**Response:** In this revised manuscript, the region of tumor area is labeled by the letter “T” in H&E staining images, and black arrows demarcate the direction of tumor cell invasion with invasive front marked by dashed line (**new Fig. 2g, 3e and 7b**). Additionally, as provided in **new Supplementary Fig. 3g, 4b and 6d**, scatter dot plots present the frequency of tumor xenografts showing invasion of tumor cells into the neighboring subcutaneous tissue in ten microscopic fields under 200× magnification, as well as the number of microscopic fields with invasive fronts between the tumor area and the neighboring subcutaneous tissue per slice of each tumor xenograft. Corresponding data have been incorporated into the revised manuscript on Pages 9, 10 and 15.

**6.** For all metastasis assays in Figure 2 and 3, did the authors attempt the orthotopic implantations followed by monitoring spontaneous metastasis?

**Response:** The model suggested by the reviewer is indeed important. We did not attempt to employ the orthotopic implantations in the initial version of the manuscript. In this revision, we evaluated the effect of overexpressing or silencing VAL on spontaneous metastasis of LAD cells using the orthotopic implantation model. As shown in **new Supplementary Fig. 3k-m**, while the growth of orthotopic xenografts of vector-control A549 or H2009 cells in the lung tissue was exclusively restricted in few lesion sites, VAL- or myr-AKT1-overexpressing LAD cells formed a number of different cancerous lesions in various lobes of the lung, as demonstrated by the extensive bioluminescent signals in the pulmonary area, and increased number of lesions of tumor cells in the slice of lung, suggesting that constitutively activated AKT1 or high levels of VAL are capable of promoting intra-pulmonary metastasis of LAD cells. By contrast, silencing VAL in H1975 cells or myr-AKT1-overexpressing LAD cells significantly suppressed intra-pulmonary metastases (**new Supplementary Fig. 4f, g**). Notably, there was no obvious evidence showing distant organ metastases in nude mice even orthotopically inoculated with LAD cells overexpressing VAL or myr-AKT1 within the 60-day experimental endpoint. This orthotopic implantation model further supports the potent pro-metastatic role of VAL in LAD progression and the crucial role of VAL in constitutively activated AKT1-driven LAD metastasis. Corresponding data have been incorporated into the revised manuscript on Pages 10 and 11.

**7.** For data related to LINC01546-Vim interactions (Figure 4-5): Overall, the data is very solid supporting the conclusions. However, authors should present a Supplementary table listing all potential LINC01546-interacting proteins identified by their mass-spec analysis. More importantly, smFISH co-staining of LINC01546 and Vim should be performed in the patient lung cancer specimens, and preferably, authors should identify lung cancers with mutant PI3K (thus increased AKT signaling) which should exhibit increased smFISH signals. For what’s presented on LINC01546 blocking Vim polyubiquitination (Figure 5h-i), the argument would be significantly strengthened if authors show similar mechanisms in patient lung tumors.

**Response:** We thank the reviewer’s comment and have taken the reviewer’s suggestion seriously. A list of all potential VAL-interacting proteins identified by the mass-spec analysis is provided as **Supplementary Table 4** in the revised manuscript. In this revision, we performed smFISH co-staining of VAL and Vimentin in clinical LAD specimens with wild-type PIK3CA or AKT and 1 case of LAD specimen with activating *PIK3CA* (E545K) mutation. As shown in **new Fig. 4h**, all of these clinical LAD specimens showed co-localization of VAL and Vimentin in the cytoplasm of lung cancer cells, and LAD specimen carrying PIK3CA activation mutation presented increased staining intensities of both VAL and Vimentin as compared to those with wild-type PIK3CA or AKT. Also importantly, in cultured primary lung cancer cells (LAD3) originated from a stage III LAD patient’s primary lung tumor, which expressed high levels of VAL, VAL was also able to bind Vimentin proteins and silencing VAL significantly suppressed protein levels other than mRNA levels of Vimentin and remarkably increased K48-linked polyubiquitination of Vimentin proteins (**new Fig. 4b, 5c and 5i**). These data strengthen the notion that the binding of VAL to Vimentin proteins abrogates polyubiquitination and degradation of Vimentin proteins and thus increases Vimentin protein levels, leading to LAD progression. Corresponding data have been incorporated into the revised manuscript on Pages 12 and 13.

**8.** For what’s shown in Figure 5a, ideally, authors should perform at least one titration or inducible experiment to show that increasing levels of LINC01546 correlate with correspondingly increased levels of Vimentin protein. Along the same line, some data in Figure 5a-g should be accompanied by quantitative analyses.

**Response:** As shown in **new Fig. 5b**, overexpression of VAL increased only protein levels of Vimentin in a dose-dependent manner, when various dosages of VAL-expressing plasmids were separately transfected into LAD cells. In parallel, using the pLVX-TRE3G-VAL Tet-on inducible system in A549 cells, addition of 2μg/ml doxycycline (Dox) for 48hr markedly induced VAL expression and upregulated protein levels, other than mRNA levels, of Vimentin (**new Supplementary Fig. 5f**). In addition, relative protein levels of immunoblots were quantified by scanning densitometry, and the relative gray value of proteins was calculated as Band Intensity of Protein of Interest / Band Intensity of Loading Control. As shown in the **new Figures 5 and 6**, the quantification values of immunoblots have been provided, which further confirms the effect of VAL on promoting Vimentin protein stabilization and inhibiting polyubiquitination and degradation of Vimentin proteins. Corresponding data have been incorporated into the revised manuscript on Pages 12 and 13.

**9.** Again for LINC01546 abrogation of Trim16-induced degradation of Vimentin (Figure 6), I would like to see similar studies performed in at least one patient lung tumor.

**Response:** In this revision, the abovementioned primary lung cancer cells LAD3 were used to validate whether VAL could abrogate Trim16-induced degradation of Vimentin. As shown in **new Fig. 6i, j**, silencing VAL failed to cause K48-linked polyubiquitination of Vimentin or decrease Vimentin protein levels when Trim16 was pre-silenced, supporting our conclusion that VAL binds to Vimentin and thus abrogates the interaction of Vimentin with its E3 ligase Trim16, resulting in disruption of Trim16-dependent degradation of Vimentin proteins in LAD cells. Corresponding data have been incorporated into the revised manuscript on Page 14.

**Minor points:**

1. Supplementary Figure 1h: the PCR band in polyA+ lane is too faint to see.

**Response:** The reviewer’s concern is well understood. In this revision, the RT-PCR assay with polyA+ and polyA-RNA fractions of LAD cells was re-performed and showed that VAL possess the 3’-terminal poly (A) structure. The image with sharp band has been provided as **new Supplementary Fig. 2d** in this revised manuscript.

2. The smFISH images in Figure 1j are not of high qualities.

**Response:** In this revision, representative images with high resolution of smFISH staining of VAL in LAD tissue and corresponding adjacent normal lung tissue, which clearly show significant upregulation of VAL levels in LAD tissue as compared to normal lung tissue and cytoplasmic localization of VAL in LAD cells, has been provided as **new Fig. 1m** in this revised manuscript.

3. The band in Figure 4f (top) is too weak.

**Response:** In this revision, the image with clearer and sharper bands of various truncated mutants of VAL as evaluated by the agarose gel electrophoresis assay has been provided as **new Fig. 4f** in this revised manuscript.

4. In Figure 6j: why siNC transfection significantly reduced Vim levels?

**Response:** We apologized for the unclear label of siLINC in the **original Figure 6j**, which should denote silencing of LINC01546, as well as for unclear description of this panel in the figure legend. In this revision, siLINC has been changed as siVAL (denotes siLINC01546) in **new Fig. 6j** and clear information has been described in the figure legend.

**5.** There are many typographical and grammatical errors throughout the manuscript. For example, Figure 2b graph (right) Y-axis should be “Cell number per field”.

**Response:** This revised manuscript has been 'spell-checked' and 'grammar-checked' carefully prior to re-submission. Appropriate corrections have been incorporated into the revised manuscript.

REVIEWERS' COMMENTS:
Reviewer#1:
In this revised manuscript, the authors have very well addressed all of my concerns. I have no additional comments.
**Response:** The authors greatly thank the reviewer for the time and the encouraging comment.

Reviewer#2:
It is a good paper, should be published.
**Response:** The authors greatly thank the reviewer for the time and the encouraging comment.

Reviewer#3:
In this extensively revised manuscript, the authors have made conscientious efforts to address my earlier comments and have substantially improved the quality of the paper. The only minor concern is about their proteomics experiments in which they identified VIM as one of the lncRNA-interacting proteins. Authors should provide a bit more experimental details on this critical experiment (e.g., did they use antisense control, any biological and/or technical replicates, any statistical treatment, etc).
**Response:** The authors greatly thank the reviewer for the encouraging comment and appreciate that the reviewer reminds us to provide more experimental details. Although the endogenous RNA pull-down assays using biotinylated short probes against VAL or its antisense for identifying VAL-interacting proteins were repeated three times, which similarly revealed distinct bands as manifested by silver staining, mass spectrometry (MS) analysis of both VAL- or VAL antisense-interacting proteins was done without biological or technical replicates. The unique peptide counts of both VAL- and VAL antisense-interacting proteins from MS analysis are provided as **new Supplementary Data 2** in this revision. Moreover, the proteins identified by MS analysis were further confirmed by endogenous RNA pull-down and Western Blots assays. Corresponding experimental details have been incorporated into the Supplementary Methods in this revision.
